# Systems engineering medicine: engineering the inflammation response to infectious and traumatic challenges

**DOI:** 10.1098/rsif.2009.0517

**Published:** 2010-02-10

**Authors:** Robert S. Parker, Gilles Clermont

**Affiliations:** 1 Department of Chemical and Petroleum Engineering, Swanson School of Engineering, University of Pittsburgh, 1249 Benedum Hall, Pittsburgh, PA 15261, USA; 2 McGowan Institute for Regenerative Medicine, University of Pittsburgh, Pittsburgh, PA, USA; 3 Department of Critical Care Medicine, University of Pittsburgh, Pittsburgh, PA, USA

**Keywords:** dynamic modelling, haemoadsorption, inflammation, sepsis, model-based control, optimization

## Abstract

The complexity of the systemic inflammatory response and the lack of a treatment breakthrough in the treatment of pathogenic infection demand that advanced tools be brought to bear in the treatment of severe sepsis and trauma. *Systems medicine*, the translational science counterpart to basic science's *systems biology*, is the interface at which these tools may be constructed. Rapid initial strides in improving sepsis treatment are possible through the use of phenomenological modelling and optimization tools for process understanding and device design. Higher impact, and more generalizable, treatment designs are based on mechanistic understanding developed through the use of physiologically based models, characterization of population variability, and the use of control-theoretic systems engineering concepts. In this review we introduce acute inflammation and sepsis as an example of just one area that is currently underserved by the systems medicine community, and, therefore, an area in which contributions of all types can be made.

## 1. Inflammation primer

Inflammation is an essential biological process that encompasses the response of pluricellular organisms to environmental stresses, such as physical damage to tissues, infection and other immune challenges. The inflammatory response seeks to avoid, contain, reverse and heal tissue damage provoked by such stresses. Inflammation is central to the pathology of major complex chronic conditions such as autoimmune diseases, arthritis, chronic lung disease, inflammatory bowel disease and psoriasis. More recently, inflammation was also linked as a major component of the pathophysiology of atherosclerosis, coronary heart disease, Alzheimer's disease (Wyss-Coray 2006), several types of cancer (Clevers 2004) and metabolic syndrome (Maiti & Agrawal 2007). Therapeutic approaches based on thwarting the inflammatory response have proven to be of great clinical benefit for many of these chronic ailments. On a different time scale, acute severe inflammatory illnesses such as sepsis, trauma and acute pancreatitis also have broad societal and economic impacts (Angus *et al.* 2001). Unfortunately, and somewhat surprisingly, modulation of inflammation has met with disappointing results for illnesses severe enough to justify admission to the modern intensive care unit (ICU) (Remick 2003).

Sepsis is a clinical syndrome representative of acute, complex inflammatory diseases. Sepsis is defined as the systemic host response to infection with clinical manifestations that span a broad set of inflammation-related signs and symptoms, such as fever, tachycardia, tachypnoea and decreased arterial blood pressure leading to clinical shock, a state of insufficient oxygen delivery to tissues characterized by hypotension and acidosis (Levy *et al.* 2003). Although the host's response to sepsis strives to contain infection and promote repair, the intensity of the inflammatory response often leads to compromised tissue function, organ failure and death. Severe sepsis accounts for 2–11% of all admissions to hospitals, approximately 750 000 cases a year, with an associated case-fatality mortality of 35 per cent (Angus *et al.* 2001). Mortality rates from sepsis and septic shock have not changed significantly over recent decades (Martin *et al.* 2003), and it affects the very young (Watson *et al.* 2003) and the very old disproportionately (Sands *et al.* 1997). The Centers for Disease Control and Prevention (CDC) reports that the incidence of sepsis increased from 7.4 per million patients in 1979 to 17.6 per million patients in 1987. Multiple organ dysfunction syndrome, associated with 80 per cent of deaths in modern ICUs (Angus *et al.* 2001), is causally related to the inflammatory response (Bone 1996), and it is also a common complication of other causes of acute severe inflammation, such as multiple trauma. The process of death typically includes progressive organ system shutdown requiring support of the circulation, ventilation and renal function.

**Figure 1.**
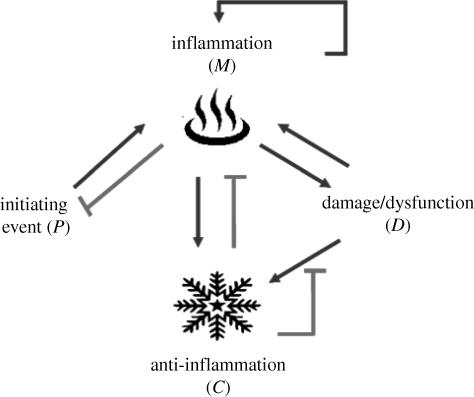
Interactions between the four components of the inflammation system. Infection (*P*) triggers inflammation (*N*). Regulatory mechanisms, conceptualized as anti-inflammation (*C*), are triggered almost simultaneously. Excessive inflammation results in tissue dysfunction (*D*), which in turn can perpetuate inflammation.

**Figure 2.**
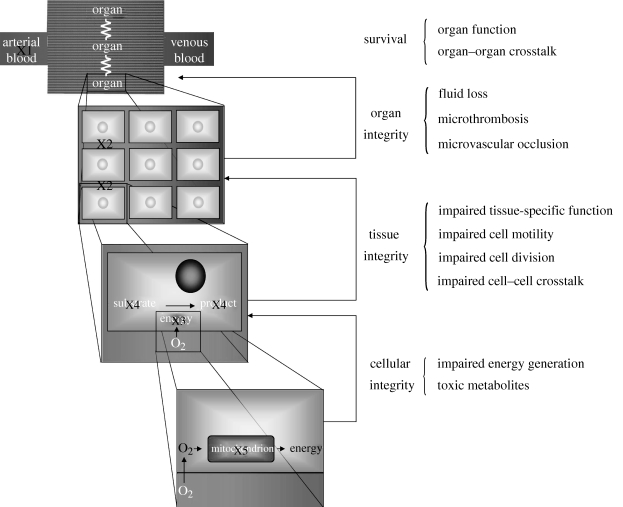
At the systemic scale, systemic inflammation decreases arterial blood pressure (X1), and therefore blood flow to organs, compromising nutrient and energy availability (see text for details). Inflammation modifies local factors (X2), further compromising the microcirculation to various tissues within organs. Tissue integrity is only possible if cells maintain their tissue-specific role, such as solute transport or metabolic function, and maintain adequate turnover and structural integrity, all of which may be compromised by inflammation-related metabolites or reprioritization of energetic resources (X3). At the lowest level, cell survival is compromised by the accumulation of toxic metabolites (X4) disrupting basic metabolism and by impaired energy production or utilization (X5).

In sepsis, acute inflammation is initiated by the recognition of danger signals using pathogen-associated molecular pattern (PAMP) receptors on the surface of dedicated surveillance cells, typically dendritic cells and tissue macrophages. Such PAMPs, associated directly to pathogens or their products (e.g. lipopolysaccharide, lipoteichoic acid, flagellin or bacterial RNA) activate dendritic cell and tissue macrophage Toll-like receptors (TLRs) (Abreu & Arditi 2004). Activation of TLRs initiates intracellular signalling of specific cascades leading to enhanced expression of early pro-inflammatory proteins, such as tumour necrosis factor (TNF) and interleukin-1 (IL-1), active in an autocrine, paracrine and endocrine fashion, with the purpose of mass mobilization of innate immunity. Regulatory anti-inflammatory proteins, such as IL-1 receptor antagonist (IL-1RA) and IL-10, almost synchronously follow. TLRs also have the ability to recognize molecular patterns, that, although not foreign, should not be accessible to those receptors in health. These damage-associated molecular patterns (DAMPs) are often the signature of cellular disruption resulting in the failure of intracellular containment of these DAMPs (Matzinger 2002). As disease progresses, it appears probable that there is perpetuation of injury, as spreading tissue damage promotes further inflammation, and inflammation contributes to tissue damage, although competing theories exist as to how this exactly happens (Prince *et al.* 2006). A pictorial representation of this dual contribution to the initiation and perpetuation of inflammation is shown in figure 1. Other important aspects of the acute inflammatory response have been described. Acute inflammation is associated with cell-type-dependent modifications of programmed cell death, appropriately retarding cell death in pathogen-fighting neutrophils, and hastening cell death in most other cell types. Inflammation promotes the production of inducible nitric oxide synthase (iNOS) leading to local vasodilatation promoting metabolite delivery and export, and key components of the complement cascade important for antimicrobial activity. Furthermore, trafficking of dendritic cells to local lymph nodes initiates specific target recognition by T-cells and clonal expansion of these cells. These steps are necessary for an appropriately controlled host response. However, the types of health challenges faced by patients in ICUs trigger inflammation at a scale unlikely to be compatible with survival, and, therefore, there is little guarantee that the evolutionary adaptation to severe infections is indeed appropriate. In other words, what is a ‘well-oiled machine’ in response to a typical challenge might not be well suited for maximal challenges. How this initial response leads to undesired effects of cellular dysfunction and organ failure remains a challenging problem from a reductionist perspective; in fact, a multi-scale systems approach may be the only fruitful approach because of the several mechanisms at different scales (figure 2) that appear to contribute to the clinical disease:

— First, an overly exuberant pro-inflammatory response can injure tissues directly. For example, the alarm-phase cytokine, TNF, is capable of inducing programmed death of functional cells in several tissues (Meldrum *et al.* 2006). Similarly, high-mobility group B1 (HMGB1), a cytokine-like protein that is released later in response to sepsis, has been shown to cause gut epithelial and hepatocellular injury (Sappington *et al.* 2002).
— Second, injury can be due to cellular hypoxia secondary to impaired tissue perfusion. Systemic inflammation causes vasodilation, increased microvascular permeability, and impaired cardiac contractility. Since flow to many vascular beds is pressure-driven below a tissue-specific threshold (Coleman *et al.* 1971), vasodilation will create inequalities in blood flow distribution with an overall decrease in blood perfusion of some tissues (through hypotension) and wasteful perfusion (through decreased resistance of the vascular bed) in other tissues. Vasodilation is thought to be caused primarily by increased production of nitric oxide (NO), which in turn is caused primarily by upregulated iNOS expression. Microvascular hyperpermeability also results from an excess of NO and various eicosanoids, as well as direct effects of TNF and IL-1. Increased permeability leads to extravasation of cell-free fluid from the circulation resulting in insufficient circulating blood volume and impaired cardiac output. Intravascular fluid loss is typically important, and preventing or correcting this loss might be a very important therapeutic action. Cardiac pump failure in sepsis is also thought, from animal experiments, to be compounded by the combined effects of TNF, IL-1 and reactive nitrogen and oxygen species (free radicals, RNS and ROS, respectively) directly altering cardiac muscular cell function (Flierl *et al.* 2008). Collectively, the effects of maldistribution of blood flow, hypovolaemia and impaired cardiac function lead to cellular hypoxia in several tissues. Since most cells depend on mitochondrial oxidative phosphorylation to generate energy, hypoxia inevitably leads to cell dysfunction or death.
— Third, activation of intravascular coagulation and thrombosis pathways may lead to organ dysfunction through thrombosis in the microcirculation. Inflammatory mediators, such as TNF, initiate the coagulation cascade (Esmon 2004), as demonstrated experimentally in large human cohorts of patients with sepsis (Dhainaut *et al.* 2003), irrespective of the causative microorganism (Kinasewitz *et al.* 2004). Inflammation also stimulates fibrinogen synthesis, further promoting coagulation. Contributing to the problem is potential occlusion of vascular beds by platelets and inflammatory cells themselves, either physically or by the formation of extra-cellular nets (Ma & Kubes 2008), the evolutionary goal of which may be to trap circulating microbial entities (Hickey & Kubes 2009).
— Fourth, processes other than hypoxia may result in cellular energetic failure. With increased NOS activity during inflammation, energy production might be impaired despite adequate intracellular PO_2_. NO directly competes with O_2_ as a substrate for respiratory chain complex III. Large quantities of intracellular NO, resulting from iNOS activity, may lead to significant competitive inhibition of electron transport, and thus inadequate ATP production. Inappropriate decreases in oxygen utilization have been described consistently under inflammatory stress. It may also be that learned mechanisms of reallocation of cellular resources under stress might be prioritizing cell survival over function in the face of limited energy supply. Oxidative stress injury could also be a major contributing mechanism to cell and organ dysfunction (Granger *et al.* 1981). According to the ‘classical model’, ischaemia leads to conversion of xanthine dehydrogenase into the enzyme xanthine oxidase (XO). In addition, during ischaemia, ATP may ultimately be degraded to xanthine. During reperfusion, O_2_ becomes available to support XO-dependent oxidation reactions that generate ROS. These free radicals, by reacting with a myriad of substrates and enzymes, interfere with normal cellular metabolism. Although ROS formation is directly related to the degree of ischaemia and the duration of hypoperfusion, its role in causing ischaemia/reperfusion (I/R) injury has been questioned (Brealey *et al.* 2004).

In summary, hypothesized and documented inflammatory response pathways interact in a complex manner, with numerous feedback loops, thereby motivating a systems-level approach to inflammation modelling. It does appear, however, that energy failure is a likely common pathway for cell dysfunction, organ failure and ultimately death.

## 2. Treating sepsis

### 2.1. Clinical successes

Modulation of immunity has led to remarkable progress in transplant medicine, where the chief objective of immune suppression of the host response to grafted immunogenic solid organs is balanced against toxic side-effects of immunosupressive regimens and the associated propensity to opportunistic infections. Years of research and observations have broadened the standard concept of immune suppression to the more subtle notion of immunotolerance. In other words, how can the immune system be fooled in accepting what is clearly non-self (Weissman & Shizuru 2008)? Several successful clinical trials have propelled the use of anti-TNF antibodies and IL-1 receptor antagonists as classes of agents for rheumatoid (Waugh & Perry 2005) and psoriatic arthritis (Punzi *et al.* 2007), as well as Crohn's disease for the former agent (Carter *et al.* 2003; Camilleri 2007), where the clinical rationale of interrupting an inflammatory loop perpetuated by an overly sensitive immune system appears sound. In addition to the well-documented side-effect of increased risk of infections, a worsening of psoriatic-like conditions (Fiorino *et al.* 2009) as well as an increased incidence of malignancies (Bongartz *et al.* 2006) have been reported, plausibly related to impaired immune surveillance (Beyer *et al.* 2009). Nevertheless, it does appear remarkable that administration of a single agent, following a pre-prescribed dosage regimen, is effective in alleviating symptoms of complex chronic inflammatory diseases where hundreds of molecular species and cell types participate in phenotypic expression, and where the body has ample time to compensate in a number of ways to both excessive TNF expression and its treatment.

### 2.2. Clinical failures

In contrast, with the exception of the proper use of antibiotics (Kumar *et al.* 2006) and human recombinant-activated protein C (Bernard *et al.* 2001), an anticoagulant with anti-inflammatory properties (Joyce & Grinnell 2002), trials at immunomodulation have largely failed for acute severe inflammatory illness, and of sepsis in particular. This failure has triggered much consternation and soul-searching in the critical care community (Marshall 2000; Sweeney *et al.* 2008). Presumably, major reasons for this dismal record relate to expectations as to how one should measure success and failure of an intervention in sepsis, and a process of insufficient reasoning in translating abundant pathophysiological knowledge acquired from extensive *in vitro* exploration and successful preclinical profiles of many lead compounds into clinically and genetically diverse human beings (Eichacker *et al.* 2002). Clinical trialists have also been obfuscated by the current burden of proof of efficacy, which still remains an improvement of all-cause mortality at a fixed time horizon, typically 28 days, rather than evidence of biological efficacy. Favourable biological activity is suggested in several trials by improvement in physiological markers (Annane *et al.* 2002; Bakker *et al.* 2004; Watson *et al.* 2004; Sharshar *et al.* 2006), typically most pronounced in the sickest patient (Bernard *et al.* 2001; Marshall *et al.* 2003; Lipiner-Friedman *et al.* 2007). As an example, a review of the first 7500 patients receiving TNF-targeting interventions suggests a significant overall benefit of 4 per cent in reducing 28-day mortality. However, no single phase III randomized clinical trial quite reached significance (Panacek *et al.* 2004). An *in silico* exploration of anti-TNF intervention in sepsis suggests that the overall mortality difference is a balance between patients truly helped by immunosupression and patients harmed by the treatment (Clermont *et al.* 2004*a*). Experimental data of the necessity of TNF for a successful response to infection (Pfeffer *et al.* 1993) support the concept that anti-TNF treatment could prevent a subgroup of patients from mounting an appropriate challenge to infection. Because of the lack of availability of reliable biomarkers for infection and the septic process, no amount of clinical data could prove harm, but this is nevertheless suggested by the severity–response relationship and *in silico* explorations. This conundrum is compounded by the nonlinear and time- and context-dependent interplay among multiple molecules, cell types, tissues and organs *in vivo*. The processes of cellular recruitment, proliferation, mode of death (necrosis versus apoptosis) and energetics manifest clinically as organ dysfunction; the complexity of this web of interactions has rendered sepsis a fertile field of often promising preclinical studies based on a reductionist approach to treatment, which failed to translate into successful therapies in actual patients. An engineer or mathematician would say that sepsis is a problem of dealing with transients: the added difficulty in the context of acute illness is that the clinician is attempting to interfere with a highly dynamic process, rather than one that has reached a ‘biological steady state’, loosely defined as one where the time scale of the intervention is much faster than that associated with disease evolution. (It should be noted that the term ‘biological steady state’ is an equilibrium state—strictly different from steady or asymptotic in the mathematical sense.) Furthermore, there is considerable variation between the onset of disease and encounter with the healthcare system; the relative contributions of variation in stage of disease at the time of encounter and individual host–pathogen idiosyncracies to clinical observations are difficult to separate. These considerations suggest that sepsis treatment, conceptualized as interfering with a dynamic process, may be more successful if titrated, and also based on a comprehensive disease model, which is currently lacking.

### 2.3. The future

The National Institutes of Health (NIH) in its Roadmap Initiative—implemented to foster transformative and cross-cutting research beyond the scope of a single institute—promotes computational modelling as a tool of knowledge discovery and possibly the preferred approach to integrate such knowledge across biological scales (http://nihroadmap.nih.gov/). Similarly, the Food and Drug Administration (FDA) in its ‘critical path’ document (Food and Drug Administration 2004) has called for the use of *in silico* models to augment preclinical studies in animals in order to develop novel therapeutic agents and devices. At present, there are no clear guidelines as to how such initiatives should be benchmarked and no external incentives to develop such initiatives within the pharmaceutical industry. Furthermore, there has been little progress in redefining or modifying the criteria of efficacy for new biologicals. The experience of the last three decades of human clinical trials, combined with a high bar for success, has resulted in fewer registrations of new randomized trials for sepsis. Therefore, the critical care community has promoted the Surviving Sepsis Campaign, a long collection of recommendations to practising clinicians as to the state-of-the-art of sepsis support and treatment. These recommendations are the result of an extensive review of existing evidence regarding diagnosis, fluid management, infection source control, antibiotic treatment and organ support for victims of severe sepsis. Remarkably absent are agents that are truly modifiers of the host response (Dellinger *et al.* 2008).

It would appear that model-based combination approaches constitute the best hope for leapfrog progress in sepsis therapy. Such approaches would clearly integrate a disease model, in the mathematical sense, existing preclinical data and base recommendations on biological readouts, such as biomarkers, which are of pathophysiological importance. Proof of concept that such an approach would shift our approach to intervention strategies and clinical trial design remains elusive for a number of legitimate reasons (Marshall 2004), including: (i) lack of a universally accepted disease model, (ii) lack of relevant data to calibrate and validate such models against, (iii) lack of biomarkers of disease activity, (iv) lack of point-of-care methods to measure such biomarkers, and (v) lack of understanding of the concept of model-based interventions by end-users. Enormous progress has been made at the basic science level, and sepsis, as a rapidly evolving disease with high case fatality, offers a unique opportunity of using systems medicine and transdisciplinary collaboration to achieve translational success.

## 3. Models for decision support

**Figure 3.**
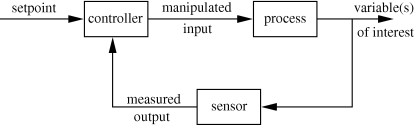
Closed-loop control schematic. Process variables of interest are measured by the sensor and compared with desired values in the controller (which includes the actuation device such as a pump or valve). Input variables are adjusted to induce changes in the outputs until the outputs reach their desired setpoint values.

As healthcare professionals, clinicians in practice make informed diagnostic and therapeutic decisions for patient care; this is implicitly based on a ‘model’ composed by the clinician's understanding of the relationships between interventions and expected outcomes of such interventions, in the context of the patient disease state. Similarly, engineers working to solve a process problem in an industrial setting use their knowledge to diagnose the problem and make appropriate changes. When feasible, this decision-making is automated via a control system or algorithm; in regulatory (setpoint tracking) mode, this system compares measurements with desired values (setpoints) and calculates the manipulated variable changes that need to be made to bring the measured quantities to their respective setpoints (figure 3). The current ‘state-of-the-art’ in the industrial setting is (non)linear model-based control, where a linear (Muske & Rawlings 1993) or nonlinear (Morari 1994; Qin & Badgwell 1999) mathematical model of the process is used explicitly in the calculation of the input changes needed to return the process to its desired operating mode. A tutorial introduction to nonlinear model predictive control can be found in Rawlings *et al.* (1994). When translating these tools to medical practice, a formal loop-closure in the engineering sense is unlikely, given that clinicians are responsible for patient safety and are justifiably hesitant to allow a computer algorithm to make a critical decision that has traditionally been made by a human. However, using a model-based algorithm to recommend a treatment intervention that is either accepted or over-ridden by a clinician may provide improvements in patient treatment by systematically exploring the potential treatment space and identifying the intervention best suited to return the individual patient to a healthy state.

An open-loop approach to treatment design and hypothesis evaluation is the focus of the PhysioLab platform of Entelos, Inc. (www.entelos.com), which uses a top-down approach to construct disease-specific *in silico* models that capture both the biology and the dynamics of the biological response. Simulation of the PhysioLabs in the areas of asthma, obesity, diabetes (types 1 and 2), rheumatoid arthritis, cholesterol metabolism, cardiovascular/atherosclerosis and skin sensitization (allergy) can be used to: (i) test a proposed intervention quickly on a simulated individual or population, (ii) hypothesize and evaluate interventions that target specific receptors or pathways, and (iii) identify potential targets for a drug or antibody intervention that may modulate the disease state. Since these studies are *in silico*, it is possible to perform a much broader and deeper analysis, for far less money and at far greater speed, than an equivalent series of studies *in vitro* or in animals.

**Figure 4. RSIF20090517F4:**
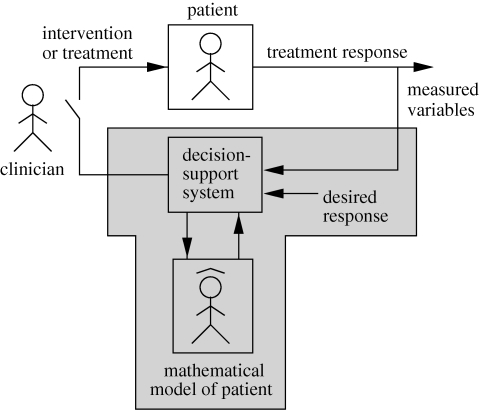
Schematic of the proposed decision-support system. Available patient measurements and their desired values are inputs to the algorithm. Systems engineering (optimization and control) tools, in concert with the patient model, establish the recommended (sub)optimal treatment intervention for the patient (shaded). A clinician verifies that the recommendation is reasonable, and, if so, the intervention is deployed to the patient (e.g. by closing the switch, left).

A critical element in this algorithmic framework is the quality of the patient model; in fact, the quality of the underlying model correlates directly with theoretically achievable performance from a model-based control system (Morari & Zafiriou 1989). The model, and its corresponding control system, take on the characteristics of the problem to some degree. In the context of a drug administration decision-support system, models of pharmacokinetics (PK; drug concentration versus time) and pharmacodynamics (PD; disease and toxicity responses to drug administration) need to be constructed. The building of such models is certainly not a new concept; PK models of drug distribution were discussed conceptually as early as 1937 (Teorell 1937*a*,*b*). An entire section below is devoted to the classes of models often employed and their respective merits/detractions. The recent advance, driven by the decrease in computer size and simultaneous increase in calculation speed, is the ability to use such models in real-time decision support, as depicted in figure 4. In the forward path lies the patient or animal undergoing treatment; this is the component for which clinicians have outstanding linear intuition. As this is often statistically motivated, the ability to alter one or two manipulated inputs, having linear effects on the outputs, would not require the level of systems understanding or computational complexity we advocate here. In contrast, consider the following: there are many (*n* > 3) measurable variables, with particular variables changing importance based on the operating (or disease) state of the patient; there are multiple potential interventions, and the effects of these inputs on clinical observables may not be a linear relationship; finally, the possible interventions may interact either positively or negatively in the determination of patient outcome. When a model is coupled with a suitable systems engineering optimization or control algorithm, a formal decision-support system may be used in a variety of modes, including simulation of candidate hypotheses and interventions as well as making treatment intervention recommendations. While the earlier reference to model quality versus performance skewed heavily in favour of developing detailed models (Morari & Zafiriou 1989), a qualification is that the ability of the model-based decision-support system to return useful (or even meaningful) recommendations is highly dependent on the appropriate complexity and structure of the underlying model, in addition to its accuracy (Parker & Doyle 2001). In other words, disease models and decision-support systems should be designed with the end-user in mind; clinical observables should be included as relevant readouts in an easy-to-use interface to the underlying computational system. Understanding some of the potential model scales, benefits and potential pitfalls is important prior to selecting a model for use in a decision-support system.

## 4. Modelling tools

A commonly employed tool, as alluded to above, is the statistically derived model based on observed associations between available measurements and an outcome, perhaps at a fixed time point (e.g. 28 day mortality). A key feature of statistical models is that predictions are based on prior observations. Consequently, such models lack sufficient predictive accuracy to provide good predictions in settings broader than those used to generate the data used in model development. This is a concept different from that of external validity, which refers to the preservation of predictive accuracy under similar experimental conditions: for example, in cohorts of patients with the same disease process, but originating from a different study. A different experimental setting would be, for example, to predict the effect of treatment from data that do not include treated patients. It is therefore outside the scope of this class of models to predict effect based solely on known mechanism of action in the absence of prior clinical observations, a deal-breaking limitation in their helpfulness in assisting the development of new therapeutic strategies. Statistical models, by construction, are designed to describe cohort behaviour and not individual behaviour. Efforts at constructing data-driven individual models have met with mitigated success and are not used in clinical practice (Chang & Bihari 1994). An additional shortcoming of most statistical models is their static nature. The dynamics of pathogenic infection and the inflammatory response cascade play a vital role in disease response, and efforts to apply more sophisticated statistical models, such as microsimulation methods, in the prediction of time-dependent outcomes, are complex and restricted to cohort behaviour (Clermont *et al.* 2004*a*).

### 4.1. Phenomenological models

Perhaps the most commonly encountered dynamic models, phenomenological models are designed to capture the observed biological response using a small number of equations and parameters. This modelling approach is ubiquitous in biomedicine: cancer chemotherapeutics are commonly modelled using linear compartmental models, using popular software packages such as ADAPT II (D'Argenio & Schumitzky 1997) and NONMEM (Sheiner & Beal 1980; Beal & Sheiner 1982; Beal 1984); in the diabetes field, the most commonly encountered model is the Bergman ‘Minimal’ Model (Bergman *et al.* 1981). The quality of this model has been debated (Quon *et al.* 1994; Finegood & Tzur 1996), but as long as its limitations are acknowledged (Weber *et al.* 1989; Steil *et al.* 1994) the original model and its extensions, including contributions from fatty acids (Roy & Parker 2006) and exercise (Roy & Parker 2007) among others, continue to be used successfully. Low-order model representations have also been employed in inflammation (see §7 for model details). The underlying structure of phenomenological models is the compartment, with each compartment represented mathematically by an ordinary differential equation (ODE), and the rates into and out of a compartment being either linear or nonlinear depending on the phenomenon requiring capture. For example, the following phenomenological model of sepsis has been previously proposed (Day *et al.* 2006*a*):
4.1
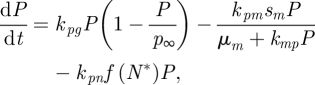

4.2
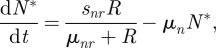

4.3
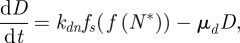

4.4
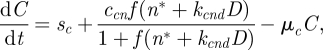


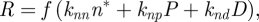

4.5
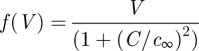

4.6
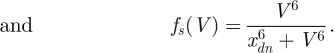



Here *P* is pathogen, N* is activated phagocytes, *D* is a marker of cellular damage and *C* is a canonical circulating anti-inflammatory mediator providing overall stability to the inflammation system. Though highly abstracted, as stated by the original authors (Day *et al.* 2006*a*), it is representative of the dynamics necessary to capture the systemic inflammatory response. Nonlinearity is a common characteristic encountered in biological systems, with saturation phenomena perhaps the most common of the nonlinear terms. Michaelis–Menten relationships are used to capture saturating effects of *P* on its own removal (second term on right-hand side of equation (4.1)) and the effect of *R* on N*. Higher order effects are captured using Hill-type nonlinearities, as in equation (4.6), which can represent two-ended saturation at both low and high *V*, as well as being used as a delay approximation when employed in dynamic equations as a function of state variables. Other common nonlinearities in biological systems include bilinearity (as in the last right-hand-side term of equation (4.1)) and inhibition, a second-order variant of which is shown in equation (4.5) where *C* downregulates the function *f*(*V*).

The shortcoming inherent in phenomenological models is captured by their name—they model the observed response or behaviour at the scale of interest, but lack mechanistic understanding and description. And while the phenomenological model structure is often chosen to simplify model parameter estimation from the available experimental data, the nonlinearities can interfere with identification as well. The calibration of parameters to experimental data is markedly easier if the dynamic model is linear in structure, although there are minimum data requirements (see Ljung 1999). Tools based on computer algebra (Audoly *et al.* 1998) have been developed to establish the property of *a priori* identifiability—the ability to uniquely identify (and quantify) all model parameters from the available experimental data. While this is a theoretical property, in that measurement uncertainty and biological variability may negatively affect quantitative accuracy of the parameter estimates, it is a valuable test that can establish the need for more measurements or a redesign of the experimental protocol prior to executing the experiment. Extending this analysis to general nonlinear systems remains an open problem, but some tools for specific nonlinearities (e.g. polynomial structures) have been developed (Audoly *et al.* 2001; Polisetty *et al.* 2006; Bellu *et al.* 2007). The challenge in both parameter calibration and *a priori* identifiability provided by ratios, common in Michaelis–Menten and Hill equations, motivates the exploration of more biologically motivated model structures. While these mechanistic descriptions are typically not linear, and of potentially higher dynamic (state) order, the ability to incorporate mechanistic information through detailed biological studies and measurements may provide an improved mapping between measurements and the model, while simultaneously assisting in the model parameter identification process. The eventual use of a model is further discussed in §5, below, but a brief foreshadow about the relationships between model structure, accuracy and utility is warranted here. When using a model to make a treatment decision, the concept of control-relevance—a model property defined as the ability to provide accurate predictions while being mathematically useful (e.g. invertible, quickly simulated) in a control or optimization algorithm—will contribute to the choice of model structure.

### 4.2. Stochastic modelling

The number of cells or molecules participating in a process that models wish to simulate may be insufficient to allow cyclical resurgence of bacterial cells (Kumar *et al.* 2004), a non-physiological physiotype permitted by the persistence of infinitesimal (≪1) quantities of microbes. In such cases, noise and stochastic effects play important roles in realistic descriptions of system behaviour. There has been a great deal of interest in applying stochastic simulation algorithms (SSAs) to cellular processes (Arkin *et al.* 1998); most of these methods rely on the Gillespie algorithm (Gillespie 1977), which is an exact simulation of a stochastic reaction process. Extensions of the Gillespie method provide the core for a number of recent simulation tools (Adalsteinsson *et al.* 2004) and methods to improve the performance of these computationally intensive methods (Rathinam *et al.* 2003). Wolkenhauer *et al.* (2004) review the advantages and disadvantages of such methods relative to the standard ODE approach outlined above. Algorithms aimed at improving the Gillespie model may still be computationally prohibitive when the number of reactions or molecules are large. In this case the net reaction rate becomes very big so that the time between events is so small that millions of steps must be taken to advance in time, with no clear prioritization of a subset of reactions. A now classic approach to this difficulty is to make an approximation in the form of the chemical Langevin equation (Gillespie 2000) (in the mathematical literature, a stochastic differential equation for Brownian motion of a particle).

Stochastic models are also important because their solution may yield qualitatively different behaviours from deterministic ODEs. For example, the ODE solution of a bistable system will evolve to either solution depending on initial conditions, while the richer stochastic solution will typically flip back and forth between the two steady states. The mean time spent in each steady state can be calculated using probability theory, and is involved in solving certain partial differential equations. Stochasticity probably plays a major role in determining which programmes will be activated in a given inflammatory cell under immunological challenge and therefore its future phenotype. Lipniacki *et al.* explored stochastic formulations of inflammation-relevant intracellular signalling (Lipniacki & Kimmel 2007) and of stochastically governed cell differentiation of T-lymphocytes (Lipniacki *et al.* 2008) in a bistable model.

### 4.3. Populations, individuals and cells, and back again

Population models are commonplace in the field of pharmacokinetics (Beal & Sheiner 1992; Sheiner & Ludden 1992; Carson & Cobelli 2001). A large number of patients, each having a small number of data points collected, are used to characterize the population average behaviour as well as the key covariates (e.g. age, race, body weight) that contribute to interpatient variability. Generally, this approach modifies a phenomenological model, like those of equations (4.1)–(4.6), by using model parameters with multiple dependencies, as follows (Sheiner & Ludden 1992):
4.7
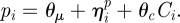



Parameters (*p*_*i*_) for patient *i* are a function of the (sub)population mean value of the parameter **θ**_**μ**_, interindividual variability in the parameter 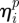, and any known correlative effects *C*_*i*_ scaled by their (sub)population mean correlation **θ**_*c*_. With sufficient data, both the underlying model structure (through the observed dynamics) and the population variability (through the need for **η** and **θ**_*c*_ or *C*_*i*_ to describe individual responses) can be characterized simultaneously. Software tools are available for constructing these models (including NONMEM (Beal & Sheiner 1992) and SPK (Resource Facility for Population Kinetics 2008)), but these tools often provide dramatically better performance with linear model structures. Given the nonlinearities often present in mechanistic representations of biological systems (especially at the cellular level for enzyme kinetics and saturations), another method may provide a more accurate model at the cellular (population) scale.

An alternative method for representing population behaviour, now in the context of cells rather than patients, is to capture the population response as a distribution, i.e. to use a population balance model (Ramkrishna 2000). The heterogeneous responses of the cells to systemic perturbations are captured through internal cellular properties (e.g. cell sensitivity, cell-cycle stage, inflammation response level, oxygen, substrate or cytokine concentration) and the definition of kernel functions. Drawbacks of this model structure are the development of the kernel functions (which are often statistical distribution driven rather than derived from biological mechanism) and its computational complexity; the common form of a population balance model is a set of partial integro-differential equations. Depending on the number of intracellular parameters and the boundary conditions, numeric solution varies from complicated to intractable (Mantzaris *et al.* 2001*a*–*c*).

Recognizing that system-level response to cellular events is composed of the collective response of individual cells, a model structure explicitly recognizing *both* the cellular model complexity and the existence of population phenomena is the cell ensemble (Domach & Shuler 1984; Shuler 1999; Henson 2003). Here, a single (intra)cellular model is specified, but the parameters of the model are recognized as coming from distributions (which are specified by the user). A population of *N*_*c*_ cells is generated via Monte Carlo sampling from the parameter distributions (Ogunnaike 2006), but with the intracellular mechanistic equation structure held constant for each cell. Overall system response is then generated by simultaneously simulating *N*_*c*_ cells. To capture intercellular dynamics and interactions with the physiological system, the cellular models are coupled to extracellular equations representing key nutrients or cytokines, similar to extracellular dynamics in population balance models (Daoutidis & Henson 2002; Zamamiri *et al.* 2002). By simulating the large number of individual cells (recognizing that the large number of equations will have an impact on model simulation speed and the ability to perform analytical analysis), the interplay between challenges (e.g. pathogenic infection) and system response at the whole-organism and cellular level, through the potentially different activation responses and cytokine production of individual cells to a stimulus, can be observed. A potential drawback of this model class lies in the difficulty in handling cell division, which may or may not be critical in models of the inflammatory response: neutrophils and macrophages, key effectors of the inflammatory response, are terminally differentiated cells (cells that do not divide); on the other hand, clonal expansion is a critical aspect of cell-mediated and humeral immunity, thought to play an important role in chronic inflammatory processes and the later phases of acute inflammation.

### 4.4. Rules-based models

There are situations where continuous quantitative models (e.g. ODEs) are less relevant because of the inability to precisely quantify state or variable values. For example, the exact peak value of circulating IL-6 may not correlate with outcome, but prolonged ‘high’ levels of IL-6 in the bloodstream may signal uncontrolled inflammation. Likewise, the ability to exactly quantify the intracellular reaction kinetics is not currently possible given the inability to measure all the necessary reactive intermediates. The reactants and products are well characterized, however. As a result of these structural uncertainties, the exact nature of quantitative models can be replaced with a more ‘fuzzy’ or rules-based approach.

Fuzzy (or fuzzy logic) modelling is used to capture imprecise or uncertain events for systems, where a precise model may be too difficult to construct or may not exist, while fuzzy control addresses the fact that human decision-making operates with approximate data and implicit objective functions (Mahfouf *et al.* 2001). In this approach, a set of tabulated rules would be constructed from the underlying physics, biology and medical understanding for the process in question. Quantitatively, variables can be classified as ‘low’, ‘medium’ or ‘high’, through the use of activation functions (conceptually similar to the formulation of neural network models, the use of mathematical formulations such as the Hill relationship or hyperbolic tangent is common). As a result, decision trees and fuzzy logic control can be used to make clinical decisions. A key advantage of this framework is the ability to specify both modelling and control (treatment) rules in clinical terms. Drawbacks include the training of models when fitting to data is required, as well as the structural selection of the underlying mathematics and solution (e.g. what is the shape of the activation function, how many levels to use for variables and interactions). It can be seen from Mahfouf and co-workers (2001) that there appears to be significant medical adoption of fuzzy modelling tools, although it can be argued whether this is a result of their ease of adoption and comprehension for clinicians or because they are the best tool for the job.

An alternative rule-based framework is the BioNetGen language (BNGL; http://bionetgen.org), a model-building paradigm that has been developed for biological systems (Blinov *et al.* 2004; Faeder *et al.* 2009). Rule specification is accomplished by defining the reactants and products with the corresponding reaction rates. The existence of specific intermediates—such as the phosphoforms along a multi-step kinase pathway or the activation of a molecule requiring multiple individual bindings to activate—need not be specified in the overall reaction, as they are handled automatically by the software in the construction of the simulation equations. While the underlying model remains of ODE or SSA types for BNGL models, the specification of such models is simplified dramatically through the rules-based engine and the simulation can be done in either deterministic or stochastic mode. A further advantage is the automated code generation by BNGL compilers, thereby reducing the typographical errors common in specifying what could be hundreds of equations for a cellular pathway or cell–cell interaction dynamics.

### 4.5. Physiological modelling

Anatomy and physiology describe, respectively, the connectivity and the physical and chemical interactions of the organs and tissues in the body. While modelling in this manner is not a new idea, in that the concept was recognized by Teorell as early as 1937 (Teorell 1937*a*,*b*), advances in computational power have made these models attractive in that they provide a greater degree of biological insight than what is available from phenomenological models. Furthermore, tissue-specific information and mechanism, when available, can be included in an intuitive and mechanistically accurate manner. Physiological models of drug pharmacokinetics are commonly employed in the cancer field (e.g. Chen & Gross 1979; Doyle *et al.* 2007; Florian *et al.* 2007), and physiological PK/PD models have been used in a variety of fields including diabetes (Sorensen 1985; Parker *et al.* 2000; Roy 2008), anaesthesiology (Wada & Ward 1994), and cancer (Jusko & Ko 1994; Florian *et al.* 2007).[Fig RSIF20090517F4]

**Figure 5.**
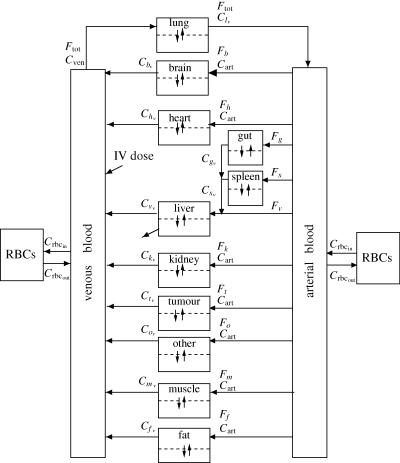
Compartment-based physiological model schematic (cancer case study—note the tumour compartment). Arrows between compartments are flows (typically blood, carrying drugs or cytokines of interest quantified as concentrations). Tissues are single compartments (well perfused) or subdivided into vascular and extravascular spaces (if significant transport resistance exists). Metabolic effects, such as clearance, DNA incorporation or irreversible intracellular binding are represented by diagonal arrows exiting particular (sub)compartments. RBC, red blood cell.

Mass balances around tissues of interest are used to construct a physiologically based model of cytokine or intervention dynamics; each tissue is therefore represented mathematically as a set of (ordinary differential) equations describing the rate of change of substances of interest within the tissue space, which can be represented as total tissue space or subdivided into vascular/extravascular (and further extravascular subdivision to interstitial/intracellular is possible). A candidate model with tissue compartments is shown in figure 5. Average values for many of the flow and volume parameters in PBPK models across multiple species are available in the literature (US EPA 1984; International Life Sciences Institute 1994). A comforting fact in using such literature parameters is the fact that circulating concentrations and measurable levels are often more sensitive to the parameters in metabolic terms than tissue flows and volumes (discussed for a diabetes example in Parker *et al.* (2000)). The associated drawback is that the remaining metabolic or reaction parameters need to be estimated from experimental data. The most commonly available data, systems-level measurements of circulating cytokines for example, can provide guidance in model specification, but these data are often insufficient to fully parameterize a physiologically based model because of failures in *a priori* identifiability—some parameters are simply not uniquely identifiable from circulating measurements. Tissue- or cellular-scale measures are often required to fully characterize this high-order model, which may require the scaling of *in vitro* experimental results to the *in vivo* scenario or the interspecies scaling of preclinical animal-derived parameters to the human patient case. Although mechanism, when identified from cellular experiments, is generally conserved, the interactions of cells with the *in vivo* environment often leads to changes in parameter values when scaling across these scenarios.

Hence, the most significant uncertainties that manifest in a physiological model involve the characterization of the intra-tissue dynamics, which are closely related to the availability and confidence in experimental measurements. As a result, the absence of tissue- or cellular-scale detail forces the modeller back to a phenomenological description of some aspect of the model. Despite this potential shortcoming, the ability to represent the body using physiological and anatomical accuracy and connectivity may still provide further benefit in understanding the system dynamics in response to disruptions. For example, a large recruitment signal for neutrophils at an injury site would not necessarily induce systemic recruitment of neutrophils throughout the body. In contrast, high circulating cytokine levels resulting in maladaptive recruitment of neutrophils or macrophages to remote uninjured sites could negatively affect the ability of the host to fight off pathogen invasion and cause undesired damage in otherwise healthy tissue. These are effects that could not result from a strictly phenomenological treatment, but can be observed in physiologically based simulations, even with incomplete mechanistic descriptions of cellular events.

## 5. Decision support tools

While mathematical models can be used to characterize and understand the dynamics of inflammation, a key reason to construct such a model is to use it explicitly in the construction of a decision-support system for real-time use in a clinical setting. Hence, an accurate model is desired (recall that model quality affects theoretically achievable performance (Morari & Zafiriou 1989)), but a model that can be efficiently and effectively employed in a systems engineering framework, known as a *control-relevant* model (Parker & Doyle 2001), is perhaps more useful. As a result, an imperfect model that captures the key observable behaviour may be the best model. Once such a model, in one of the frameworks discussed in the previous section, is constructed, the following tools can be used to design treatment interventions.

### 5.1. Optimization and optimal control

Optimal control is a tool commonly described in the literature in the solution of biomedical systems problems. Here an objective is coupled with a set of constraints, and the entire problem is solved either analytically or via programming techniques. Optimal control problems are mathematically posed as follows (Bertsekas 1995):
5.1
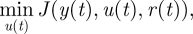

subject to
5.2
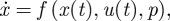

5.3
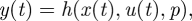

5.4
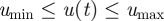

5.5
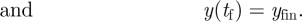

While the objective, *J*, is general, the most commonly employed engineering objective function (equation (5.1)) is the least-squares deviation of a variable of interest, *y*(*t*), from the user-supplied target trajectory, *r*(*t*). This minimization (or equivalently maximization if the objective is multiplied by −1) is performed over a time horizon from 0 (taken as the present time, by convention) to *t*_f_, a user-specified final time. The optimization variable is *u*(*t*), which can be independently specified over the entire horizon.

The constraints are typically model descriptions, incorporated as ODEs (equations (5.2) and (5.3)), and the acceptable bounds on the optimization variable (equation (5.4)) are either explicitly determined from physical constraints (e.g. pump rates, non-negativity) or implicit limits resulting from physical phenomena (e.g. flow rate limits imposed by haemolysis). Finally, an endpoint for the variable(s) of interest is specified, as in equation (5.5).

As an example, consider a haemoadsorption device that would be used to remove cytokines from the blood. An optimal control problem might have the following specification. The target values (*r*(*t*)) could be the basal cytokine levels, and the model-predicted values would be cytokine levels from a mathematical model, which are compared with actual patient-specific values using point-of-care measurements (about an hour delayed from real-time for measurement processing). Manipulating blood flow through the haemoadsorption device would alter the rate of cytokine removal by the device, and, as such, the concentration of cytokines at the device exit (where blood is returned to the body). The lower bound on flowrate, the optimization variable, would be just greater than the flow at which coagulation occurs in the device; the upper bound is the point just before blood haemolyses in the device. Finally, a desired end-of-haemoadsorption-treatment cytokine level would be specified (again, perhaps the basal levels). The result is an optimal control problem for cytokine regulation via haemoadsorption in response to sepsis.

Solving this problem is equivalent to solving a two-point boundary value problem. Under a set of assumptions, analytical solutions are available. However, a programming solution is more common, and a commonly employed tool is control vector parameterization (Martin & Teo 1994). This involves a discretization of the time axis into *k* equal-length segments, over which the optimization variable(s) (e.g. blood flow rate through the device) is held constant, and the levels of *u*(*k*) are the decision variables in the resulting optimization problem. The exact method for solving this optimization is up to the user; gradient search is perhaps the most common. The input profile generated by the optimization often has a characteristic ‘bang-bang’ shape, where the input profile over the horizon of interest switches back and forth between the applied constraints, *u*_min_ and *u*_max_. This technique is good for solving nonlinear control problems with constraints, but there are inconsistencies with medical practice—primarily, there is no accommodation made for real-time feedback adjustment of the treatment profile in the presence of new measurement data (that are collected while the optimal control-generated therapy is being administered).

### 5.2. Receding horizon control

An alternative approach to model-based treatment intervention is to use receding horizon, or model predictive, control (Muske & Rawlings 1993; Allgöwer *et al.* 1999). This formulation poses and solves an open-loop optimization each time a measurement is collected, the result of which is a sequence of input values or changes to be implemented over a user-specified time horizon. Structurally, the problem is similar to the optimal control formulation above; a cytokine or damage-associated objective is minimized (again, typically a sum-of-squared-error goal (5.6)) over a horizon, haemoadsorption flowrate (or other intervention) is held constant between measurements, models are used explicitly in the algorithm solution (5.7 and 5.8), and constraints on the inputs can be rigorously enforced (5.9 and 5.10). The departure from an optimal control formulation comes in two places: (i) the endpoint constraint is not included and (ii) the problem is reposed and the solution recalculated each time a measurement is collected. A typical model predictive control (MPC) problem may be formulated as follows (Muske & Rawlings 1993; Morari & Ricker 1994):
5.6
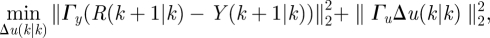

subject to
5.7
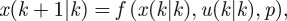

5.8
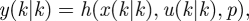

5.9
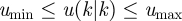

5.10
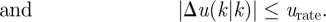



The mathematical formalism employs statistical notation; 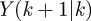 is the vector of predicted measurable outputs (length *N*_*p*_) at time *k* + 1 given information up to time *k*. The desired trajectories or values for the cytokines or damage surrogate(s) is 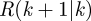, and the optimization variables (the degrees of freedom for the optimization) are the input *changes* 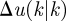 (length *N*_*m*_). The *change* in the input, rather than the input value, is commonly employed in the MPC formalism to help eliminate steady-state offset, a condition where *Y* ≠ *R* at stable operation. Trade-offs between the need to keep measured cytokines or other outputs near their desired values, versus minimizing the undesired effect of small variations in the measurement (due to measurement variability inherent to the measurement device or method) altering the intervention, are accomplished by altering the weighting matrices 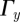 and 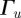, respectively.

**Figure 6.**
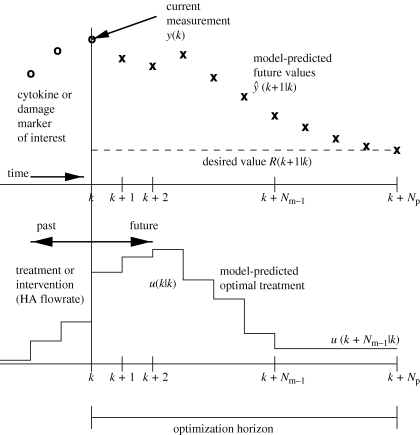
Receding horizon (model predictive) control implementation. Minimizing the deviation of model-predicted outputs (e.g. cytokine concentrations, crosses) from the desired reference value(s) (dashed line) is accomplished by implementing input changes (e.g. haemoadsorption device flow rate, solid line). Solution schematic is for ‘time *k*’, which will be updated and resolved at ‘time *k* + 1’ (the next measurement time).

As in optimal control, an optimization routine is called in the solution of the MPC problem, and, as a result, input constraint incorporation is straightforward in both magnitude-constrained (equation (5.9)) and rate-constrained (equation (5.10)) forms. Output and state constraints may lead to infeasibilities in the optimization problem, and as a result these are often omitted or incorporated in a ‘soft’ form by replacing the hard constraint with corresponding penalty terms in the objective function (Zafiriou & Chiou 1993). A schematic of MPC implementation is shown in figure 6.

Revisiting the example from §5.1, at the present time *k*, the current cytokine levels are measured and compared with the model-predicted cytokine values. To address (expected) differences between the patient and the model, an additive correction (*d*(*k*)) is made to the model predictions over the *N*_*p*_-length prediction horizon. The difference between the desired cytokine values (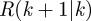) and the model-predicted ones (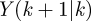) over the *N*_*p*_-length prediction horizon is used to calculate the *N*_*m*_-length series of haemoadsorption flow rate changes that minimize the objective (typically driving *Y* as close as possible to *R*). The first flow rate change is implemented, possibly requiring confirmation from a clinician, and the process repeats when the next measurement is collected.

In the case of time-variable, or temporally mismatched, measurements of different entites (e.g. cytokines and damage surrogates), a multi-rate MPC formulation is available (Lee *et al.* 1992; Gopinath *et al.* 1995). Here, the algorithm makes decisions using model-predicted values until new measurement information is available, with each measured quantity updated independently. While the MPC problem formally returns a solution that is suboptimal, in that it is a locally optimal solution resolved at each measurement time point, the algorithm has shown excellent performance and a respectable degree of robustness in the industrial application setting (Qin & Badgwell 1999). The structural advantages of MPC, including the on-line solution of an optimization problem that incorporates available patient information, are significant, but a key drawback of this control structure is the challenging analysis of algorithm performance. Algorithm stability guarantees, which may be required by the FDA before deployment in a clinical setting, often require the use of endpoint constraints or long prediction horizons (i.e. large *N*_*p*_ or ‘infinite-horizon’ formulations (Rawlings *et al.* 1994)), which may limit the achievable performance.

### 5.3. Mixed-integer programming

To this point, the modelling and control theoretic tools have focused on ODEs and continuous-valued variables. However, some measurements (e.g. DNA arrays and other colorimetric methods) provide a less numerically precise estimate. Alternatively, a modeller may choose not to use real-valued variables (because of experimental uncertainty or atypical natural distributions), but rather to discretize the variables as ‘high’ or ‘low’. ODE-based models are poorly posed to handle this qualitative information because these lumped models are founded on the continuum assumption. This is the same reason that ODE models do not work well for small (i.e. countable) numbers of molecules—the continuum assumption loses validity in this regime.

In a similar manner, treatment or outcome variables are not necessarily continuous. Most common are the ‘yes/no’ type—the decision to treat or not to treat, response or no response, survival or death. These are examples of binary [0, 1] variables, yet highly relevant in the clinical setting. Oral drug administration is often explicitly or implicitly quantified, in the manner of fixed-dose pills (e.g. one aspirin or two?) or infused drugs mixed at specific concentrations or dose levels. Treatment decisions are also made on quantified observations, such as ‘high’, ‘moderate’ or ‘low’ levels of TNF. Rather than resorting to fuzzy logic, mixed-integer programming (MIP) is a candidate formal mathematical structure for handling these discrete values. Here discrete quantities are assigned corresponding integer values, so increasing doses might be represented as levels 1, 2 and 3, for example, with no treatment assigned a value of zero. If all variables were discrete, an integer programming problem would result; this further reduces to a binary program if all variables—not just the decision variables—are integer-valued in the range [0, 1]. However, drug dose levels or other quantified interventions interact with continuous biosystems models, in general; an administered drug dose results in a PK profile that is continuous. This continuously valued information is the ‘mixed’ part of the MIP problem. Based on the user-selected objective, and the structure of the underlying system model, the nature of the MIP problem is specified: a linear objective and model yields a mixed-integer linear programming (MILP) problem; replacing the objective with a quadratic form, as in the MPC problem above (equation (5.6)), yields an MIQP (Q for quadratic); and the explicit inclusion of other nonlinear terms (e.g. Michaelis–Menten or Hill saturations, product inhibition) requires the solution of an MINLP (NL for nonlinear).

Consistent with clinical practice, and using the MPC problem in equations (5.6)–(5.10) as an example, it is possible to repose this problem to explicitly address quantified information using a MIQP or MINLP problem (Harrold & Parker in press), depending on the system model used, as follows:
5.11
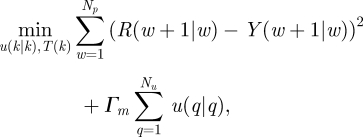

5.12
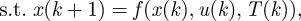

5.13
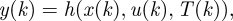

5.14
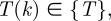

5.15
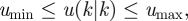

5.16
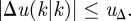



The receding-horizon control problem independent variables, 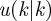, and the integer-valued decision variables, *T*(*k*), are used to minimize the objective function (5.11). The additional equations represent discrete-time model states (5.12) and outputs (5.13), the quantized variable treatment definition (5.14), continuous input bounds (5.15) and rate of change constraints (5.16), as well as integer variable Big-M constraints (not explicitly shown). An advantage of this formulation is that new patient information can be incorporated as a measurement update and an improved solution computed. While such an approach appears daunting, experience with simulated cancer treatments indicates that clinically relevant problems (1894 equations having 865 continuous and 168 discrete variables) can be solved within a second on a desktop computer (Harrold & Parker 2009).

## 6. Issues in modelling biological processes

Constructing plausible descriptive or predictive models of biological processes faces issues that are commonly not encountered in physics/engineering models, or are generally less severe. Sources of uncertainty and variability are routinely encountered in biological data, the notion of the existence of biological laws is on much softer grounds, and the very concept of the existence of clear causal chains loses clarity in complex systems with numerous redundancies, feedback mechanisms and levels of regulatory control. Impressive advances in data acquisition and processing techniques have and will continue to help alleviate some of these obstacles, while others await further advances in information, mathematical and systems theory.

### 6.1. Variability, uncertainty, similarities and differences

Whether a biological process exists or how it actually happens are often significant sources of uncertainty. The concept is quite intuitive when applied to the existence of the exact wiring of specific signalling pathways, where elegant specific solutions typically requiring extensive experimental–theoretical collaborations have helped (Kuepfer *et al.* 2007). Some of the core processes involved in the pathophysiology of sepsis are clearly multi-scalar, however—hypotension causes organ dysfunction in a number of ways. Cellular energy failure is undoubtedly a major driver of cellular dysfunction, but inability to clear toxic metabolites or direct organ–organ crosstalk, in the form of mechanical or neural interactions between organs themselves, may also play major roles. The modelling implication of uncertain mechanisms and their relative importance, as broadly described above in the pathophysiology of acute inflammatory diseases and sepsis, is that the process of designing disease models should not be limited to the construction of a single model. Rather, the focus should be the inclusion and evaluation of competing hypotheses, as often done in weather forecasting (Gneiting & Raftery 2005), or some balanced representation of such hypotheses according to some prior likelihood (obtained from the existing empirical evidence or expert opinion) embodied in a consensus model. At the present time, the examination of competitive models has been mostly restricted to machine-learning approaches to competitive wiring of signalling pathways. Couched in dynamical systems language, these factors translate to uncertainty in model structure or rules, and, within a given structure, to uncertainty in parametrization. Compounding the problem for processes with unclear starting points, such as sepsis, uncertainty in the initial conditions and variation in experimental conditions must also be considered. This is particularly disconcerting when controlled experimental conditions result in wide inter-individual variability of measurements across cohorts of genetically identical mice (a common observation in preclinical cancer chemotherapy studies). These two factors may in fact be crucial to our lack of success in translating promising preclinical information into actionable therapies in humans (Salluh & Bozza 2008; Dyson & Singer 2009).

Measurement uncertainty, both in accuracy (reproducibility of data given identical circumstances) and bias (how close is the measurement to the true value), is typically of greatest severity in biological data. For example, several fluorescent antibody-based assays, such as enzyme immunosorbent-linked assays (ELISA) or multiplex bead-based capture techniques (e.g. Luminex) measure fluorescence units and assume either that the binding reaction between the fluorescent antibody and molecule of interest has reached equilibrium or that the degree of completion of the binding reaction is exactly known at the time of the readout. Transposition of the fluorescence value to a standard curve yields the desired measurement, which is typically an average between (often very different readouts of) duplicates or triplicates. In our experience, we often had to reconstruct such standard curves and adapt them to our specific experimental conditions. Reproducibility and standardizations of experimental protocol and measurements remain significant sources of variability that modellers must take into account in their model calibration efforts. Thus, interventions can range from reappraising data, as described above, to the construction of models that take into account such variability in the process of structure and parameter estimation in a statistical or probabilistic fashion (Bertrand *et al.* 2001; Zenker *et al.* 2007). In spite of such caveats, there is also remarkable regularity in the inflammatory response across individuals and across species. The responses to acute endotoxin administration in rodents and humans are quite similar in their timing and sequence of mediators activated, although rodents are typically very resistant to endotoxin, requiring a dose per weight higher by several orders of magnitude to create comparable physiological response (Boujoukos *et al.* 1993; Chow *et al.* 2005). Therefore, the wiring structure of the acute inflammatory response is essentially robustly defined. That differences across individuals and species rest both in actual relative importance of participating mechanisms and in the difficulty to fully appreciate variations in experimental conditions offers hope that model-based approaches can constructively deal with biological uncertainty and resolve conflicting reports inhibiting progress in translation.

### 6.2. Stochasticity and randomness

Even genetically identical organisms do not respond identically to equivalent challenges. The variance observed in data collected from animal studies is, to some degree, a function of the measurement device and method. However, this does not capture the entirety of the spread observed. While this is one of the driving factors behind the use of statistical methods in the design and analysis of animal and human trials, it also calls into question the use of ODEs in the modelling of population average data—or more accurately, the benefits and drawbacks of constructing such models. An advantage of this simplified model structure at the whole-organism level is that uncertainties need not be captured mechanistically at every level of detail (e.g. organ, cell, nucleus); instead, these can be lumped into parameters characterizing the observed data at the organism scale. The corresponding drawback is that the loss of mechanistic accuracy may inherently limit the utility of the average ODE model in the treatment design for an individual (Morari & Zafiriou 1989).

As discussed above, one method for handling this variability is in the context of population models (Beal & Sheiner 1992; Sheiner & Ludden 1992; Resource Facility for Population Kinetics 2008). Here, the variability is incorporated into an ODE model as parameter variability, with the model parameters belonging to distributions informed from (pre)clinical data. As the resolution is increased to organs, cells and intracellular dynamics, the ability to inform population-style models from data decreases because of the general lack of reliable *in vivo* measurements at the smallest scales. Furthermore, the continuum assumption may begin to break down as properties such as heterogeneity, well-mixedness and large numbers of molecules may be violated. At highly resolved scales, interactions are not deterministic—the *probability* of a meaningful interaction is dependent, using molecular reactions as example, on the reactant molecules, energy and perhaps enzyme availability. Although the mechanistic structure of the (intra)cellular process may still be unknown, this variability can be incorporated at the correct scale through the use of stochastic ODEs or other appropriately abstracted stochastic simulators (see §4.2, above, for more detail on the modelling methods).

### 6.3. Collaboration between experimentalists and theoreticians

It is interesting to note from discussions with academics and industrial PhDs the degree to which academic control theory research has *not* made the translation to the industrial sector. Rigorous tools that provide performance guarantees, such as robust control and control systems designed using Lyapunov stability analysis, are not widely employed in the chemical process industry. Likewise, nonlinear control is a powerful tool, but its use has also been limited to those applications clearly requiring its capabilities (i.e. where linear control tools are insufficient on performance or stability grounds) and also of a state dimension to admit solution. In contrast, the model predictive control paradigm has been highly successful in industry and enjoys wide acceptance. However, the studies of robust performance guarantees and the necessary mathematical tools again do not often translate beyond the academic realm. The reason for this translation failure is at least twofold: (i) a control system that requires exclusive expertise to *maintain* after deployment is judged ‘too expensive’ unless the industry has a *very* high margin (e.g. pharmaceuticals) and (ii) the solution to the control problem is both theoretically complex, making it hard for the non-expert to understand the need for such a system, and computationally complex, such that the completion of the calculations cannot be guaranteed in a real-time industrial setting. These obstacles extend beyond the chemical industry.

Engineers and mathematicians have been addressing biomedical problems for decades. In diabetes, the first models were published in the early 1960s (Bolie 1961), with the first truly successful translation of model to clinic being the ‘Minimal Model’ by Bergman and co-authors in 1981 (Bergman *et al.* 1981). Likewise, cancer models date back at least to the 1970s, and only in the 2000s have model-derived treatment decisions started to manifest (Fornier & Norton 2005). Interesting to note in both of the clinical successes cited here is that the primary author is a medical doctor. Both Dr Bergman and Dr Norton have worked over the years with mathematicians and engineers (Prof. C. Cobelli being the engineer and driving force in the diabetes modelling area; he has continued to work on this problem (Nucci & Cobelli 2000; Bellazzi *et al.* 2001; Cobelli *et al.* 2007; Pedersen *et al.* 2008)). In contrast, the plethora of papers published by engineers and mathematicians claiming to have ‘solved’ such problems as glucose control, HIV treatment, cancer therapy and anaesthesia delivery—among other examples—address only the ‘necessary’ part of the problem. In other words, a technological solution has been presented that, if provided suitable information, can successfully achieve a stated objective. The missing piece is the clinical and translational aspect, the ‘sufficient’ component of a necessary and sufficient condition, where a clinician would participate in posing the objective and constraints of the problem to make sure that a proposed solution is sufficient for deployment in a clinical setting. As the case study below will demonstrate, the collaboration of clinicians with engineers and mathematicians leads to mathematical models that are both efficient (in their use of states, parameters, etc.) and allow the evaluation of clinically relevant questions. Furthermore, the ability to use these mathematical models in the context of treatment design, or their *control relevance* (Parker & Doyle 2001), is a design criterion as well, as discussed above.

## 7. A case study in inflammation

### 7.1. Low-order approximants

Our initial attempt to model the acute inflammatory response comprised a very high level, three-variable model: a growing pathogen (*P*); a pathogen predator (*M*) that multiplies in the presence of pathogen and destroys pathogen; and a late inflammatory mediator (*L*) triggered by *M* and which can itself grow (Kumar *et al.* 2004). The system of ODEs below represents this process where the *k*'s are rate constants and **θ** and *W* are form factors for the hyperbolic tangent function
7.1
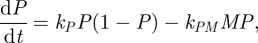

7.2
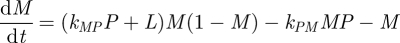

and
7.3
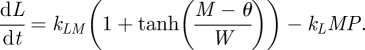



Analysis of this system of equations reveals the possibility of a number of intricate behaviours, ranging from elimination of *P* and resolution of both *M* and *L* (health), to elimination of *P*, but persistence of *M* and *L* (aseptic sepsis), to persistence of all three variables at high levels (septic sepsis), to persistence of *P*, but low persistent levels of *M* and *L* (immunosupression), to cyclic behaviour. All behaviours result over different ranges of initial conditions (dose of *P*) and system parameters. Lessons from this low-order model include the possibility of identifying conditions for the existence of self-sustaining inflammation and the possibility of parameter and initial condition-dependent existence of tristability (Kumar *et al.* 2004). As a first extension of this model, the inflammatory response is in fact the result of a balance between effectors that amplify the inflammatory cascade and a parallel anti-inflammatory response effect by counter-regulatory mediators that prevents the pro-inflammatory cascade from getting out of hand, so that a minor local infection does not result in a massive mobilization of resources. Active counter-regulation is a widespread feature of acute biological response mechanisms, blood coagulation being another prominent example (Adams *et al.* 2007). In abstract form, the three-state model of acute inflammation is extended by the introduction of an anti-inflammatory mediator (*C*) and by modifying the meaning of *L* to represent tissue damage (*D*) (and thus a marker of health/death)—these changes characterize a variety of four-variable models (Day *et al.* 2006*a*; Reynolds *et al.* 2006) (see also figure 1). These were also formally analysed mathematically and can give rise to a wide range of behaviours. Simulations explored the impact of modulating exogenous administration of *M* or *C* on the asymptotic level of *D*. In other words, manipulations of the immune response will change the trajectory and final state of the system (Day *et al.* 2006*b*; Reynolds *et al.* 2006).

Our first attempt at calibrating a model of acute inflammation was performed with a rather large (22-equation) model (Chow *et al.* 2005). This work introduced the hypothesis that acute inflammation was using the same mechanisms irrespective of the specific stressor causing inflammation. Because a single model with a unique set of parameters was used to describe the inflammatory response to endotoxin, trauma caused by a laparotomy and haemorrhagic shock, we believe that this model provides strong support for a universal wiring of the acute phase of the inflammatory response. All experiments in support of the model were conducted in mice. Serum TNF, IL-6, IL-10 and nitrites/nitrates were measured at four time points within a 24 h period following the insult. Because the experiments were conducted in mice, animals had to be sacrificed at each time point, and therefore no longitudinal time series were obtained from individual animals. Furthermore, only four of the 22 state variables were measured, resulting in a highly under-determined model. Contrary to a widespread misconception, even severely under-determined models such as this one cannot be forced to fit the data if the mechanisms embodied in the equations are biologically unsound (mathematically this would be interpreted as a dynamic structural mismatch between the data and the model formulation). Finally, we obtained a calibration of the model by convex optimization. Using a standard local/convex optimization routine such as Newton–Raphson or Levenberg–Marquardt will identify, from a starting guess of parameter values, a set of parameters that minimizes the error between the simulated model and observed data. Such algorithms will typically converge to a local minimum, which will yield different model parameterizations if different initial guesses are used. Indeed, as a result of the underspecified and underconstrained local optimization, it is typically possible to produce many candidate models that fit the empirical data acceptably well, while (possibly) possessing very different behaviours in unobserved states, or simply relying on different mechanistic drivers to produce the same results. In the case presented, there is undoubtedly a large collection of parameter sets that also could have fitted the dataset (Chow *et al.* 2005). This limitation does not invalidate the conclusion of universality of the acute inflammatory response, but affects the potential application of this particular parameter set to predictions of subsequent experiments if those were to be substantially different. In other words, this naïve approach lacks robustness and carries a high risk of the model not translating to subsequent experiments.

**Figure 7.**
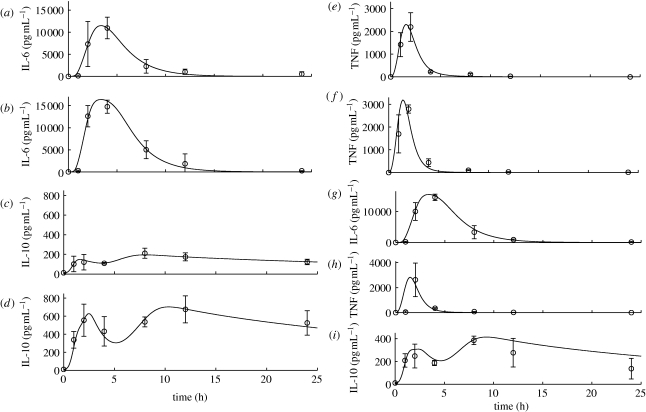
Model simulations and experimental rat endotoxaemia data. IL-6 model calibration results from (*a*) 3 and (*b*) 12 mg kg^−1^ endotoxin challenge. IL-10 model calibration results from (*c*) 3 and (*d*) 12 mg kg^−1^ endotoxin challenge. TNF model calibration results from (*e*) 3 and (*f*) 12 mg kg^−1^ endotoxin challenge. *Model predictions* versus experimental data for (*g*) IL-6, (*h*) TNF and (*i*) IL-10 at an endotoxin challenge level of 6 mg kg^−1^.

We were not satisfied with the ratio of observed variables to actual model variables, and with the relative complexity of the models. Therefore, we scaled back to the minimum meaningful number of variables that could still describe the data; conceptually akin to the successful ‘Minimal’ modelling approach used by Bergman and Cobelli for diabetes (Bergman *et al.* 1981). The result was a generation of models that have between eight and 10 variables, including pro-inflammatory mediators TNF and IL-6, anti-inflammatory mediator IL-10, serum neutrophil counts and serum creatinine, a marker of organ (renal) dysfunction (Daun *et al.* 2008). Calibration was performed on doses of endotoxin of 3 mg kg^−1^ and 12 mg kg^−1^ administered to 250 g Sprague–Dawley rats, four per dose, with seven time points per animal over 24 h. A prediction was provided for an intermediate dose of 6 mg kg^−1^, as shown in figure 7 (Daun *et al.* 2008).

One challenge in model calibration is the solution of the least-squares problem for parameter estimation. We have adopted a systematic approach to solving this inverse problem.

— First, constrain parameter space by constructing lucid bounds on parameter values. This is often possible from literature and (pre)clinical data/knowledge.
— Second, construct a set of heuristics that penalize parameter sets for producing model behaviours that are deemed unrealistic. For example, models that fail to ‘heal’ in finite time with negligible stimulation or display peaks in unobserved variables of unacceptable/unbelievable duration or magnitude lead to further parameter space constraints.
— Third, we proceeded to explore formal model parameter reduction techniques (Daun *et al.* 2008) (not to be confused with model reduction techniques, which generally focus on reduction of the state—not parameter—dimension (Moore 1981; Glover 1984; Chiang & Safonov 1992)). This process involves calculating the sensitivity of the simulation results to small changes in parameter values (Yue *et al.* 2006). The analysis is typically performed for each variable in the model at each time point where data are available, although we are also applying methods that perform global sensitivity (Carter *et al.* 2003; Bongartz *et al.* 2006). We then proceed to an iterative process of fixing parameters to which the model is insensitive and recalculating sensitivities. This process of parameter reduction is stopped when there are no more insensitive parameters. The ‘price to pay’ is then evaluated by recalibrating the model only using sensitive parameters. This analysis also yields another extremely important piece of information: the process of identifying sensitive parameters, on the one hand, and the difficulty of constraining them through literature searches, on the other, identify where critical knowledge gaps exist. It was clear from our modelling efforts that simulations were extremely sensitive to pathogen burden and rate of growth, and also to the specific distribution of immune cells. Experimentally, we had virtually no information on either of those domains of data. Consequently, the outlook for building truly predictive models of sepsis to explore pathophysiological hypotheses remains grim until longitudinal quantification of key drivers are better described experimentally or clinically.
— Finally, given animal variability and data uncertainty, there are legitimate reasons to carry a large number of parameter sets as representative for our animal population. Accordingly, our consensus model is actually an ensemble of models, obtained in one of the two ways: (i) multi-start convex optimization of the reduced model, where the ensemble may include several hundred model parameterizations (Waugh & Perry 2005), or (ii) a fully probabilistic approach where parameter space is explored using Markov chain Monte Carlo algorithms (e.g. Metropolis-Hastings (Bertrand *et al.* 2001; Zenker *et al.* 2007)) and parameter sets are given a probability density based on their ability to fit experimental data.

This approach, in our estimate, represents the most satisfactory solution to the inverse problem, and may also be the most practical in terms of providing probabilistic predictions (Hancioglu 2007).

The observation that acute inflammation is compartmentalized is of high clinical significance (Boujoukos *et al.* 1993). Compartmental ODEs are quite useful when modelling processes involving tissue localization or transport across membranes and when the number of elements belonging to each species (e.g. molecules of a specific protein) is large, but where spatial processes such as diffusion are not critical. We are currently working on a systematic process for developing such models and provide an example here. Using a reduced model that we developed for sepsis (Reynolds 2008) as the starting point, we created models with varying numbers of compartments, typically two or more tissue compartments communicating via a blood compartment. These models included pathogen, macrophages and neutrophils as cell types, a pro-inflammatory effector, an anti-inflammatory effector and tissue damage. Macrophages are confined to the tissue, while the rest of the components are diffusible. In particular, neutrophils diffuse into compartments following pro-inflammatory gradients (see fig. 20 and subsequent in Reynolds (2008)). Simulations are initiated by seeding one or more organs with pathogen and admit a minimum of three fixed points, as do simple models: restoration of health; high pathogen, high damage death; and no pathogen, high damage death. Typically, linked organs either heal together or die together. Simulations suggest that relative tissue volumes, relative diffusivity and resident macrophage populations are strong determinants of outcome. Compartmentalization of the inflammatory response may also solve apparent contradictions observed in the literature, including situations where animals dying of a severe infection mount an appropriate response to infection at the primary site, while inflammatory cells are diverted to peripheral tissues, and why interventions not primarily directed at the source may help improve outcomes (Alves-Filho *et al.* 2005).

### 7.2. A case study in therapeutics: blood purification for sepsis

**Figure 8.**
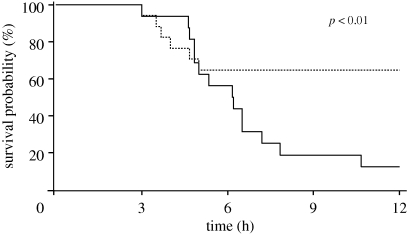
Twelve-hour post-intervention mortality with (dashed lines) and without (solid lines) haemoadsorption.

The Cytosorb blood purification device consists of polystyrene di-vinyl benzene copolymer beads of 300–800 µm diameter in a biocompatible coating packed in a 10 g cartridge with a total surface area of 850 m^2^ g^−1^. When inserted in parallel with the animal's circulation starting 20 h after cecal ligation and puncture (an inflammatory insult resulting in native bacteria invading the peritoneal cavity) for a duration of 4 h, we observed a significant improvement in short-term mortality (figure 8), mortality at one week (data not shown) and a remarkable clearance of TNF, IL-10 and IL-6, arguably key cytokines in the early pathophysiology of sepsis. Blood purification is a feasible intervention in humans, and optimizing the conditions under which a blood purification device should be used seems a desirable goal. One can also assume that those conditions, like the titrated care offered by an ICU physician, would vary dynamically and thus could be represented in the form of a sophisticated systems engineering problem, if a suitable model of sepsis, and of the control intervention, were to exist.

**Figure 9.**
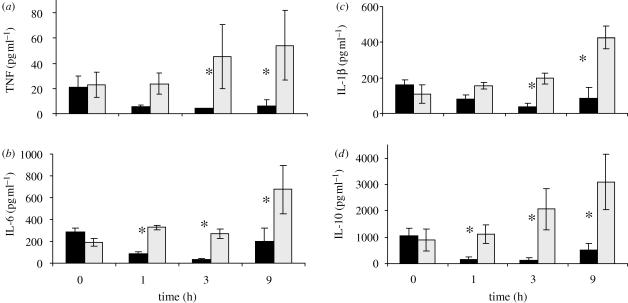
Clearance of (*a*) TNF, (*b*) IL-6, (*c*) IL-1β and (*d*) IL-10 by the Cytosorb blood purification device. Asterisk indicates *p* < 0.05 between control and treated groups.

To characterize cytokine-specific ability of the bead material to adsorb a molecule, cytokine-rich serum was filtered through the column *ex vivo* (Dileo *et al.* 2009). Other parameters characterizing the intervention are either known (e.g. bead mass and density) or are intervention dependent (e.g. timing and duration of intervention, blood flow). The blood purification intervention model shown in figure 9 can be coupled to a compartmental model ensemble, for evaluating individual response to the treatment intervention. This last step is the current work in progress.

## 8. Discussion

### 8.1. Key clinical needs and barriers to adoption of methodology

There has been considerable focus on methods to improve the difficult and costly process of validating the positive impact of new interventions on outcomes in phase III randomized clinical trials (Annane 2009). The need for such an approach is particularly urgent in the field of acute inflammation secondary to trauma and sepsis (Yaffe & Fink 2000; Cobb *et al.* 2005). As systems scientists attempt to bridge the gaps between massive data streams offered by high-throughput methods and the existing analytical methods, and to develop a coherent multi-scale framework of interpretation, the need to prioritize data and computational methods that will help accomplish this goal is essential (Ye *et al.* 2005). A significant effort is therefore present to articulate concepts underpinning the discipline of systems medicine, as a translationally relevant extension of systems biology (Auffray *et al.* 2009).

A number of obstacles along this path remain. First, there is a perceived dichotomy between the traditionally inductive approach of the scientific method and the deductive approach of model-based hypothesis generation (Kitano 2002). In fact, there is no such dichotomy—they are complementary, in that model predictions, obtained from simulations, must still be subjected to experimental verification. Still, there is no consensus as to what constitutes a reasonable burden of proof that a prediction is actually verified, while there is the intuition that it may not always be possible to reduce the problem of prediction validity, and thus model validity, to the *p*-value of a statistical test. Second, clinicians and scientists not trained in quantitative methods, which are ultimately responsible for completing the bench to bedside realization, are typically agnostic of many of the quantitative methods discussed above. This is unlikely to change, and thus efforts at promoting interdisciplinary exchange and collaborations should be encouraged. Furthermore, the tools developed, potentially through such collaborations, should be packaged to facilitate the practice of medicine and treatment—potentially a tall order for what is otherwise ‘research-grade’ code that requires an expert to execute. Third, there are no sufficiently detailed *in silico* models of human sepsis because of: (i) existing residual uncertainty in the causal chain of outcomes, thus yielding uncertainty in model structure and measurement accuracy and bias, (ii) large gaps in data at relevant modelling scales to calibrate such models, (iii) lack of availability of point-of-care testing of relevant analytes, and (iv) theoretical and algorithmic deficiencies in the existing methods of maximizing information derived from incomplete and disparate data. The support of organizations such as the International Society for Systems Biology (http://www.issb.org), the Society for Complexity in Acute Illness (http://www.scai-med.org), several national funding agencies and private foundations to articulate these difficulties, to promote communication across disciplines and importantly to maintain a focus on the translational relevance of this effort are commendable.

### 8.2. On translating animal results, which are not human, to humans

The study of specific biological mechanisms of sepsis, especially if cell type specific, is best done *ex vivo* under controlled conditions. When the objective is to vary entire host conditions (e.g. knock-out genes) or interfere with a disease's natural course (e.g. therapeutic attempts), then one has to resort to *in vivo* disease models. Animal models of sepsis are still too heterogeneous with regard to type of insult, duration and supportive therapy to be regarded as representative of the human condition. Using standardized animal models may eliminate some of the differences between animal and human studies, allowing a greater degree of translation (Dyson & Singer 2009). There have been attempts at standardizing animal models of sepsis and septic shock over the last two decades (Fink 1990). Many will argue that caecal ligation and puncture, where the colon is ligated at the hepatic flexure and the caecum perforated with a needle allowing bacterial efflux into the abdomen, represents a reasonable experimental construct of sepsis (Buras *et al.* 2005; Fink 2008; Nemzek *et al.* 2008). However, the advantages of such an animal disease model are balanced by the fact that human sepsis is obviously more heterogeneous, with several potential local sources of infection (pneumonia, renal system, abdominal, etc.).

Although the basic wiring of the acute inflammatory response appears very similar across species, the relative contribution of the myriad processes may be very different. For example, the lung has a high resident macrophage population to aid in infection clearance and it is also a highly perfused organ. Therefore, the lung is easily inflamed when compared with other organs, whether it is the primary site of infection or not. One would then expect sepsis therapies to have differential success depending on the primary site of infection, which has been confirmed clinically (Bernard *et al.* 2001). Adding to the difficulty is that timing of therapy is critical in a rapidly evolving disease, also eloquently demonstrated in sepsis (Rivers *et al.* 2001; Bernard *et al.* 2004). Unfortunately, time zero of an infection is truly not known in a typical patient, in contrast to an animal experiment. Some effort has to be put into biomarker-based outcome stratification algorithms or estimation routines that provide calculated projections of time and magnitude of initial infection. Such strategies are promising in animal series (Osuchowski *et al.* 2009), and also in human cohorts (Kellum *et al.* 2007; Rivers *et al.* 2007), in support of previous *in silico* work (Clermont *et al.* 2004*b*).

Animal models that have demonstrated efficacy have typically done so by a demonstrable reduction of an extremely high mortality in otherwise healthy animals, where the cause of death was obviously attributable to sepsis. In humans, however, the baseline mortality is lower and, given the demographics of the target population, sepsis is a contributing factor to mortality rather than the unique cause. Therefore, the extrapolation of animal studies to humans could well be improved if less lethal animal models were used, preferably in older animals. Genetic factors also play an important role in both the risk of sepsis and its outcome, and several polymorphisms have been associated with either susceptibility or outcome (Kellum 2003). Factors such as sex and race also influence susceptibility and severity of sepsis, plausibly through complex genetic factors (Dombrovskiy *et al.* 2007; Dyson & Singer 2009). Only recently have animal experiments focused on sex-specific markers and outcomes. While there is progress in understanding the underlying physiological correlates of age and sex, there is no clear parallel to the notion of race in animals. Within species, strains is a vastly more radical concept than that of race. Therefore, the translation from basic science in animals to clinical outcomes in humans at present remains a difficult proposition. Careful planning of future animal experiments, in concert with intensive efforts at developing mechanistic computational disease models that include known physiological correlates such as age and sex, may offer the best chance at correctly predicting outcomes and the impact of specific interventions based on biomarker profiles in humans.

### 8.3. Systems medicine: how systems approaches drive advances in clinical practice

The systems biology boom has resulted in the continuing generation of tremendous amounts of published data (a 10/2009 search for ‘systems biology’ returned more than 10 000 hits on www.pubmed.org), as well as systems-derived analysis tools for addressing this complex dataset (representative reviews include Doyle & Stelling (2006); Polpitiya *et al.* (2009)). At the heart of this field is the collaboration between basic science, in this case biology, and systems science, including engineering, mathematics and computer science. But what about translational science? The application of biological knowledge to improve the human condition is the practice of medicine; hence, *systems medicine* is the coupling of systems science with medical treatment decision-making (Auffray *et al.* 2009). This field is borne of the recognition that disease diagnosis and treatment is a systems response problem, incorporating multiple inputs, measurements and unobservable effects, and that simple one-pathway or one-effect methods are unlikely to succeed on a broad scale. The key advance observed over the past 10–15 years that opened the possibility of translating systems medicine to the bedside is the transition of mathematical and engineering approaches to clinical problems from ‘theoretically interesting’ (and generally devoid of concrete clinical practice knowledge) (Martin 1992; Parker *et al.* 1999; Zurakowski & Teel 2006) to practicable methods that explicitly incorporate clinical knowledge and constraints in diseases such as cancer (Lee & Zaider 2008; Harrold & Parker 2009), diabetes (Bondia *et al.* 2009; Dua *et al.* 2009; Kumareswaran *et al.* 2009) and glucose control in critical care (Plank *et al.* 2006; Hovorka *et al.* 2008; Blaha *et al.* 2009; Cordingley *et al.* 2009).

From a systems engineering perspective, the field of systems medicine is rife with challenges. Model structures are neither fixed nor mechanistically characterized in many cases, so model selection and reconciliation with data from multiple studies (and collection methods) is needed. Measurements and analytical methods have inherent uncertainty in the reported values, meaning that parameter calibration and model structure selection have conflicting objectives of accuracy and robustness to small perturbations. An advantage to having a high-fidelity mathematical model, however, is the ability to use simulation as an analysis tool—examples include: parametric and model structure sensitivity analysis (Huang *et al.* 2001; Yue *et al.* 2006); evaluation of current ‘standard of clinical practice’ to the base model and any parametric or structural perturbations; *a priori* identifiability analysis and experimental design (Audoly *et al.* 1998, 2001; Bellu *et al.* 2007); and the development of ‘what if’ scenarios via Monte Carlo methods (for perturbations) or for the evaluation of novel intervention strategies. The development of model-based treatment decision-support systems relies heavily on the underlying model (Morari & Zafiriou 1989; Parker & Doyle 2001), thereby asking for both predictive accuracy and robustness when the model is used to make decisions as part of a formal algorithm such as model predictive control. Unfortunately, performance and robustness are properties that must often be balanced (Skogestad & Postlethwaite 1996), so it is important to involve clinicians when tuning algorithm response to achieve clinically desired performance and robustness levels. To address the theoretical challenges, in the context of the variety of possible clinical case studies, is a challenge to the theoretical and clinical participants of the transdisciplinary collaborations advancing systems medicine.

## References

[RSIF20090517C1] Abreu M. T. Arditi M. 2004 Innate immunity and toll-like receptors: clinical implications of basic science research. J. Pediatr. 144, 421–429. (10.1016/j.jpeds.2004.01.057) 15069387

[RSIF20090517C2] Adalsteinsson D. McMillen D. Elston T. C. 2004 Biochemical network stochastic simulator (bionets): software for stochastic modeling of biochemical networks. BMC Bioinformatics 5, 24 (10.1186/1471-2105-5-24) 15113411 PMC408466

[RSIF20090517C3] Adams G. L. Manson R. J. Turner I. Sindram D. Lawson J. H. 2007 The balance of thrombosis and hemorrhage in surgery. Hematol. Oncol. Clin. North Am. 21, 13–24. (10.1016/j.hoc.2006.11.013) 17258115

[RSIF20090517C4] Allgöwer F. Badgwell T. A. Qin J. S. Rawlings J. B. Wright S. J. 1999 Nonlinear predictive control and moving horizon estimation: an introductory overview. Advances in control—highlights of ECC '99, pp. 391–449. London, UK: Springer.

[RSIF20090517C5] Alves-Filho J. C. Benjamim C. Tavares-Murta B. M. Cunha F.Q. 2005 Failure of neutrophil migration toward infectious focus in severe sepsis: a critical event for the outcome of this syndrome. Mem. Inst. Oswaldo Cruz. 100(Suppl. 1), 223–226. (10.1590/S0074-02762005000900038) 15962127

[RSIF20090517C6] Angus D. C. Linde-Zwirble W. T. Lidicker J. Clermont G. Carcillo J. Pinsky M. R. 2001 Epidemiology of severe sepsis in the United States: analysis of incidence, outcome, and associated costs of care. Crit. Care Med. 29, 1303–1310. (10.1097/00003246-200107000-00002) 11445675

[RSIF20090517C7] Annane D. 2009 Improving clinical trials in the critically ill: unique challenge–sepsis. Crit. Care Med. 37(Suppl. 1), S117–S128. (10.1097/CCM.0b013e318192078b) 19104211

[RSIF20090517C8] Annane D. 2002 Effect of treatment with low doses of hydrocortisone and fludrocortisone on mortality in patients with septic shock. JAMA 288, 862–871. (10.1001/jama.288.7.862) 12186604

[RSIF20090517C9] Arkin A. Ross J. McAdams H. H. 1998 Stochastic kinetic analysis of developmental pathway bifurcation in phage lambda-infected *Escherichia coli* cells. Genetics 149, 1633–1648. 9691025 10.1093/genetics/149.4.1633 PMC1460268

[RSIF20090517C11] Audoly S. D'Angio L. Saccomani M. P. Cobelli C. 1998 Global identifiability of linear compartmental models: a computer algebra algorithm. IEEE Trans. Biomed. Eng. 45, 36–47. (10.1109/10.650350) 9444838

[RSIF20090517C10] Audoly S. Belu G. D'Angio L. Saccomani M. P. Cobelli C. 2001 Global identifiability of nonlinear models of biological systems. IEEE Trans. Biomed. Eng. 48, 55–65. (10.1109/10.900248) 11235592

[RSIF20090517C12] Auffray C. Chen Z. Hood L. 2009 Systems medicine: the future of medical genomics and healthcare. Genome Med. 1, 2 (10.1186/gm2) 19348689 PMC2651587

[RSIF20090517C14] Bakker J. 2004 Administration of the nitric oxide synthase inhibitor ng-methyl-l-arginine hydrochloride (546c88) by intravenous infusion for up to 72 hours can promote the resolution of shock in patients with severe sepsis: results of a randomized, double-blind, placebo-controlled multicenter study (study no. 144-002). Crit. Care Med. 32, 1–12. (10.1097/01.CCM.0000105118.66983.19) 14707554

[RSIF20090517C17] Beal S. L. 1984 Population pharmacokinetic data and parameter estimation based on their first two statistical moments. Drug Metabol. Rev. 15, 173–193. (10.3109/03602538409015064) 6745081

[RSIF20090517C15] Beal S. L. Sheiner L. B. 1982 Estimating population kinetics. CRC Crit. Rev. Biomed. Eng. 8, 195–222. 6754254

[RSIF20090517C16] Beal S. L. Sheiner L. B. 1992 NONMEM user's guide. NONMEM Project Group. San Francisco, CA: University of California.

[RSIF20090517C18] Bellazzi R. Nucci G. Cobelli C. 2001 The subcutaneous route: closed-loop and partially closed-loop strategies in insulin dependent diabetes mellitus. IEEE Eng. Med. Biol. 20, 54–64. (10.1109/51.897828) 11211661

[RSIF20090517C19] Bellu G. Saccomani M. P. Audoly S. D'Angio L. 2007 DAISY: a new software tool to test global identifiability of biological and physiological systems. Comp. Meth. Prog. Biomed. 88, 52–61. (10.1016/j.cmpb.2007.07.002) PMC2888537 17707944

[RSIF20090517C20] Bergman R. N. Phillips L. S. Cobelli C. 1981 Physiologic evaluation of factors controlling glucose tolerance in man. J. Clin. Invest. 68, 1456–1467. (10.1172/JCI110398) 7033284 PMC370948

[RSIF20090517C21] Bernard G. R. 2001 Efficacy and safety of recombinant human activated protein C for severe sepsis. N. Engl. J. Med. 344, 699–709. (10.1056/NEJM200103083441001) 11236773

[RSIF20090517C22] Bernard G. R. Margolis B. D. Shanies H. M. Ely E. W. Wheeler A. P. Levy H. Wong K. Wright T. J. 2004 Extended evaluation of recombinant human activated protein C United States trial (Enhance US): a single-arm, phase 3b, multicenter study of drotrecogin alfa (activated) in severe sepsis. Chest 125, 2206–2216. (10.1378/chest.125.6.2206) 15189943

[RSIF20090517C23] Bertrand C. Ohmi M. Suzuki R. Kado H. 2001 A probabilistic solution to the meg inverse problem via MCMC methods: the reversible jump and parallel tempering algorithms. IEEE Trans. Biomed. Eng. 48, 533–542. (10.1109/10.918592) 11341527

[RSIF20090517C24] Bertsekas D. P. 1995 Dynamic programming and optimal control, vols 1 and 2 Belmont, MA: Athena Scientific.

[RSIF20090517C25] Beyer M. Steinhoff M. Anagnostopoulos I. Assaf C. Sterry W. 2009 Hepatosplenic T-cell lymphomas and therapy with TNF-alpha-blocking biologics: a risk for psoriasis patients? J. Dtsch. Dermatol. Ges. 7, 191–194. (10.1111/j.1610-0387.2008.06961.x) 19192165

[RSIF20090517C26] Blaha J. 2009 Comparison of three protocols for tight glycemic control in cardiac surgery patients. Diabetes Care 32, 757–761. (10.1007/s00134-008-1236-z) 19196894 PMC2671097

[RSIF20090517C27] Blinov M. L. Faeder J. R. Goldstein B. Hlavacek W. S. 2004 BioNetGen: software for rule-based modeling of signal transduction based on the interactions of molecular domains. Bioinformatics 20, 3289–3291. (10.1093/bioinformatics/bth378) 15217809

[RSIF20090517C28] Bolie V. W. 1961 Coefficients of normal blood glucose regulation. J. Appl. Physiol. 16, 783–788. 13870789 10.1152/jappl.1961.16.5.783

[RSIF20090517C29] Bondia J. Dassau E. Zisser H. Calm R. Vehí J. Jovanovic L. Doyle F. J. III 2009 Coordinated basal-bolus infusion for tighter postprandial glucose control in insulin pump therapy. J. Diabetes Sci. Technol. 3, 89–97. 20046653 10.1177/193229680900300110 PMC2769848

[RSIF20090517C30] Bone R. C. 1996 Immunologic dissonance: a continuing evolution in our understanding of the systemic inflammatory response syndrome (SIRS) and the multiple organ dysfunction syndrome (MODS). Ann. Intern. Med. 125, 680–687. 8849154 10.7326/0003-4819-125-8-199610150-00009

[RSIF20090517C31] Bongartz T. Sutton A. J. Sweeting M. J. Buchan I. Matteson E. L. Montori V. 2006 Anti-TNF antibody therapy in rheumatoid arthritis and the risk of serious infections and malignancies: systematic review and meta-analysis of rare harmful effects in randomized controlled trials. JAMA 295, 2275–2285. (10.1001/jama.295.19.2275) 16705109

[RSIF20090517C32] Boujoukos A. J. Martich G. D. Supinski E. Suffredini A. F. 1993 Compartmentalization of the acute cytokine response in humans after intravenous endotoxin administration. J. Appl. Physiol. 74, 3027–3033. 8366003 10.1152/jappl.1993.74.6.3027

[RSIF20090517C33] Brealey D. Karyampudi S. Jacques T. S. Novelli M. Stidwill R. Taylor V. Smolenski R. T. Singer M. 2004 Mitochondrial dysfunction in a long-term rodent model of sepsis and organ failure. Am. J. Physiol. Regul. Integr. Comp. Physiol. 286, R491–R497. (10.1152/ajpregu.00432.2003) 14604843

[RSIF20090517C34] Buras J. A. Holzmann B. Sitkovsky M. 2005 Animal models of sepsis: setting the stage. Nat. Rev. Drug Discov. 4, 854–865. (10.1038/nrd1854) 16224456

[RSIF20090517C35] Camilleri M. 2007 Anti-TNF antibodies for Crohn's disease. N. Engl. J. Med. 357, 1662 (10.1056/NEJMc072381) 17942882

[RSIF20090517C36] Carson E. Cobelli C. (eds) 2001 Modelling methodology for physiology and medicine. San Diego, CA: Academic Press.

[RSIF20090517C37] Carter J. D. Valeriano J. Vasey F. B. 2003 Crohn disease worsened by Anakinra administration. J. Clin. Rheumatol. 9, 276–277. (10.1097/01.RHU.0000081265.06408.e4) 17041471

[RSIF20090517C38] Chang R. W. Bihari D. J. 1994 Outcome prediction for the individual patient in the ICU. Unfallchirurgica 97, 199–204. 8197466

[RSIF20090517C39] Chen H. S. Gross J. F. 1979 Physiologically based pharmacokinetic models for anticancer drugs. Cancer Chemother. Pharmacol. 2, 85–94. (10.1007/BF00254079) 93986

[RSIF20090517C40] Chiang R. Y. Safonov M. G. 1992 Robust control toolbox. Natick, MA: The Mathworks, Inc.

[RSIF20090517C41] Chow C. C. 2005 The acute inflammatory response in diverse shock states. Shock 24, 74–84. (10.1097/01.shk.0000168526.97716.f3) 15988324

[RSIF20090517C42] Clermont G. 2004 *a* Dynamic microsimulation to model multiple outcomes in cohorts of critically ill patients. Intens. Care Med. 30, 2237–2244. (10.1007/s00134-004-2456-5) 15502934

[RSIF20090517C43] Clermont G. 2004 *b* *In silico* design of clinical trials: a method coming of age. Crit. Care Med. 32, 2061–2070. 15483415 10.1097/01.ccm.0000142394.28791.c3

[RSIF20090517C44] Clevers H. 2004 At the crossroads of inflammation and cancer. Cell 118, 671–674. (10.1016/j.cell.2004.09.005) 15369667

[RSIF20090517C45] Cobb J. P. 2005 Application of genome-wide expression analysis to human health and disease. Proc. Natl Acad. Sci. USA 102, 4801–4806. (10.1073/pnas.0409768102) 15781863 PMC555033

[RSIF20090517C46] Cobelli C. Toffolo G. M. Dalla Man C. Campioni M. Denti P. Caumo A. Butler P. Rizza R. 2007 Assessment of beta-cell function in humans, simultaneously with insulin sensitivity and hepatic extraction, from intravenous and oral glucose tests. Am. J. Physiol. Endocrinol. Metab. 293, E1–E15. (10.1152/ajpendo.00421.2006) 17341552

[RSIF20090517C47] Coleman T. G. Granger H. J. Guyton A. C. 1971 Whole-body circulatory autoregulation and hypertension. Circ. Res. 28(Suppl. 87), II76–II87. 10.1161/01.res.28.5.ii-76 5568235

[RSIF20090517C48] Cordingley J. J. 2009 Intensive insulin therapy: Enhanced model predictive control algorithm versus standard care. Intens. Care Med. 35, 123–128. 10.1007/s00134-008-1236-z 18661120

[RSIF20090517C50] Daoutidis P. Henson M. A. 2002 Dynamics and control of cell populations in continuous bioreactors. In Proc. CPC VI, *Tucson, AZ, January 2001.* AIChE Symposia Series. Austin, TX: CACHE Corporation.

[RSIF20090517C49] D'Argenio D. Z. Schumitzky A. 1997 ADAPT II users guide: pharmacokinetic and pharmacodynamic systems analysis software. Los Angeles, CA: Biomedical Simulations Resource, University of Southern California.

[RSIF20090517C51] Daun S. Rubin J. Vodovotz Y. V. Roy A. Parker R. S. Clermont G. 2008 An ensemble of models of the acute inflammatory response to bacterial lipopolysaccharide in rats: results from parameter space reduction. J. Theor. Biol. 253, 843–853. 18550083 10.1016/j.jtbi.2008.04.033

[RSIF20090517C53] Day J. Rubin J. Vodovotz Y. Chow C. C. Reynolds A. Clermont G. 2006 *a* A reduced mathematical model of the acute inflammatory response. II. Capturing scenarios of repeated endotoxin administration. J. Theor. Biol. 242, 237–256. (10.1016/j.jtbi.2006.02.015) 16616206

[RSIF20090517C52] Day J. D. Rubin J. Florian J. Parker R. P. Clermont G. 2006 *b* Modulating inflammation using nonlinear model predictive control. J. Crit. Care 21, 349–350.

[RSIF20090517C54] Dellinger R. P. 2008 Surviving sepsis campaign: international guidelines for management of severe sepsis and septic shock: 2008. Intens. Care Med. 34, 17–60. (10.1007/s00134-007-0934-2) PMC2249616 18058085

[RSIF20090517C55] Dhainaut J. F. 2003 Drotrecogin alfa (activated) (recombinant human activated protein c) reduces host coagulopathy response in patients with severe sepsis. Thromb. Haemost. 90, 642–653. 14515185 10.1160/TH02-11-0270

[RSIF20090517C56] Dileo M. V. Kellum J. A. Federspiel W. J. 2009 A simple mathematical model of cytokine capture using a hemoadsorption device. Ann. Biomed. Eng. 37, 222–229. (10.1007/s10439-008-9587-8) 18949559 PMC2758484

[RSIF20090517C57] Domach M. M. Shuler M. L. 1984 A finite representation model for an asynchronous culture of *E. coli.* Biotech. Bioeng. 26, 877–884. (10.1002/bit.260260810) 18553472

[RSIF20090517C58] Dombrovskiy V. Y. Martin A. A. Sunderram J. Paz H. L. 2007 Occurrence and outcomes of sepsis: influence of race. Crit. Care Med. 35, 763–768. (10.1097/01.CCM.0000256726.80998.BF) 17255870

[RSIF20090517C59] Doyle F. J. III Stelling J. 2006 Systems interface biology. J. R. Soc. Interface 22, 603–616. (10.1098/rsif.2006.0143) PMC1664650 16971329

[RSIF20090517C60] Doyle F. J. III Seborg D. Parker R. S. Bequette B. W. Jeffrey A. Xia X. Craig I. McAvoy T. J. 2007 A tutorial on biomedical process control. J. Proc. Cont. 17, 571–594. (10.1016/j.jprocont.2007.01.012)

[RSIF20090517C61] Dua P. Doyle F. J. III Pistikopoulos E. N. 2009 Multi-objective blood glucose control for type 1 diabetes. Med. Biol. Eng. Comput. 47, 343–352. (10.1007/s11517-009-0453-0) 19214613

[RSIF20090517C62] Dyson A. Singer M. 2009 Animal models of sepsis: why does preclinical efficacy fail to translate to the clinical setting? Crit. Care Med. 37(Suppl. 1), S30–S37. (10.1097/CCM.0b013e3181922bd3) 19104223

[RSIF20090517C63] Eichacker P. Q. 2002 Risk and the efficacy of antiinflammatory agents: retrospective and confirmatory studies of sepsis. Am. J. Respir. Crit. Care Med. 166, 1197–1205. (10.1164/rccm.200204-302OC) 12403688

[RSIF20090517C64] Esmon C. T. 2004 Crosstalk between inflammation and thrombosis. Maturitas 47, 305–314. (10.1016/j.maturitas.2003.10.015) 15063484

[RSIF20090517C65] Faeder J. R. Blinov M. L. Hlavacek W. S. 2009 Rule-based modeling of biochemical systems with BioNetGen. Methods Mol. Biol. 500, 113–167. 19399430 10.1007/978-1-59745-525-1_5

[RSIF20090517C66] Finegood D. T. Tzur D. 1996 Reduced glucose effectiveness associated with reduced insulin release: an artifact of the minimal–model method. Am. J. Physiol. 271, E485–E495. 8843742 10.1152/ajpendo.1996.271.3.E485

[RSIF20090517C67] Fink M. P. 1990 Leaky gut hypothesis: a historical perspective. Crit. Care Med. 18, 579–580. 2328605

[RSIF20090517C68] Fink M. P. 2008 Animal models of sepsis and its complications. Kidney Int. 74, 991–993. (10.1038/ki.2008.442) 18827799

[RSIF20090517C69] Fiorino G. Allez M. Malesci A. Danese S. 2009 Review article: anti-TNF-alpha induced psoriasis in patients with inflammatory bowel disease. Aliment. Pharmacol. Ther. 29, 921–927. (10.1111/j.1365-2036.2009.03955.x) 19210297

[RSIF20090517C70] Flierl M. A. Rittirsch D. Huber-Lang M. S. Sarma J. V. Ward P. A. 2008 Molecular events in the cardiomyopathy of sepsis. Mol. Med. 14, 327–336. 18256728 10.2119/2007-00130.Flierl PMC2227904

[RSIF20090517C71] Florian J. A. Jr 2007 A physiologically-based pharmacokinetic (PBPK) and pharmacodynamic model of docetaxel (Doc) and neutropenia in humans. In American Society of Clinical Oncology Annual Meeting, Chicago, IL, June 2007. Alexandria, VA: American Society of Oncology.

[RSIF20090517C72] Food and Drug Administration. 2004 Innovation or stagnation: challenge and opportunity on the critical path to new medical products. Technical report. See http://www.fda.gov/downloads/ScienceResearch/SpecialTopics/CriticalPathInitiative/CriticalPathOpportunitiesReports/ucm113411.pdf.

[RSIF20090517C73] Fornier M. Norton L. 2005 Dose-dense adjuvant chemotherapy for primary breast cancer. Breast Cancer Res. 7, 64–69. (10.1186/bcr1007) 15743513 PMC1064124

[RSIF20090517C74] Gillespie D. T. 1977 Exact stochastic simulation of coupled chemical reactions. J. Phys. Chem. 8, 2340–2361. (10.1021/j100540a008)

[RSIF20090517C75] Gillespie D. T. 2000 The chemical langevin equation. J. Chem. Phys. 113, 297–306. (10.1063/1.481811)

[RSIF20090517C76] Glover K. 1984 All optimal hankel norm approximations of linear multivariable systems, and their l_∞_-error bounds. Int. J. Control 39, 1145–1193. (10.1080/00207178408933239)

[RSIF20090517C77] Gneiting T. Raftery A. E. 2005 Atmospheric science: weather forecasting with ensemble methods. Science 310, 248–249. (10.1126/science.1115255) 16224011

[RSIF20090517C78] Gopinath R. Bequette B. W. Roy R. J. Kaufman H. Yu C. 1995 Issues in the design of a multirate model-based controller for a nonlinear drug infusion system. Biotechnol. Prog. 11, 318–332. (10.1021/bp00033a013) 7619401

[RSIF20090517C79] Granger D. N. Rutili G. McCord J. M. 1981 Superoxide radicals in feline intestinal ischemia. Gastroenterology 81, 22–29. 6263743

[RSIF20090517C80] Hancioglu B. 2007 Mathematical modeling of virus dynamics in immunology. PhD thesis, Department of Mathematics, University of Pittsburgh, PA. See http://etd.library.pitt.edu/ETD/available/etd-12072007-105848/unrestricted/07Handissertation.pdf.

[RSIF20090517C81] Harrold J. M. Parker R. S. 2009 Clinically relevant cancer chemotherapy dose scheduling via mixed-integer optimization. Comput. Chem. Eng. 33, 2042–2054.

[RSIF20090517C82] Henson M. A. 2003 Dynamic modeling of microbial cell populations. Curr. Opin. Biotechnol. 14, 460–467. (10.1016/S0958-1669(03)00104-6) 14580574

[RSIF20090517C83] Hickey M. J. Kubes P. 2009 Intravascular immunity: the host-pathogen encounter in blood vessels. Nat. Rev. Immunol. 9, 364–375. (10.1038/nri2532) 19390567

[RSIF20090517C84] Hovorka R. Chassin L. J. Ellmeer M. Plank J. Wilinska M. E. 2008 A simulation model of glucose regulation in the critically ill. Physiol. Meas. 29, 959–978. (10.1088/0967-3334/29/8/008) 18641427

[RSIF20090517C85] Huang H. P. Lee M. W. Chen C. L. 2001 A system of procedures for identification of simple models using transient step response. Ind. Eng. Chem. Res. 40, 1903–1915. (10.1021/ie0005001)

[RSIF20090517C86] International Life Sciences Institute. 1994 Risk Science Institute Working Group on physiological parameters. Physiological parameter values for PBPK models. A report prepared by the International Life Sciences Institute, Risk Science Institute under a cooperative agreement with the US Environmental Protection Agency, Office of Health and Environmental Assessment.

[RSIF20090517C87] Joyce D. E. Grinnell B. W. 2002 Recombinant human activated protein C attenuates the inflammatory response in endothelium and monocytes by modulating nuclear factor-kappab. Crit. Care Med. 30(Suppl. 5), S288–S293. (10.1097/00003246-200205001-00019) 12004250

[RSIF20090517C88] Jusko J. W. Ko H. C. 1994 Physiologic indirect response models characterize diverse types of pharmacodynamic effects. Clin. Pharmacol. Ther. 56, 406–419. 7955802 10.1038/clpt.1994.155

[RSIF20090517C89] Kellum J. A. 2003 Hemoadsorption therapy for sepsis syndromes. Crit. Care Med. 31, 323–324. (10.1097/00003246-200301000-00060) 12545045

[RSIF20090517C90] Kellum J. A. 2007 Understanding the inflammatory cytokine response in pneumonia and sepsis: results of the genetic and inflammatory markers of sepsis (genims) study. Arch. Intern. Med. 167, 1655–1663. (10.1001/archinte.167.15.1655) 17698689 PMC4495652

[RSIF20090517C91] Kinasewitz G. T. 2004 Universal changes in biomarkers of coagulation and inflammation occur in patients with severe sepsis, regardless of causative micro-organism. Crit. Care 8, R82–R90. (10.1186/cc2459) 15025782 PMC420030

[RSIF20090517C92] Kitano H. 2002 Systems biology: a brief overview. Science 295, 1662–1664. (10.1126/science.1069492) 11872829

[RSIF20090517C93] Kuepfer L. Peter M. Sauer U. Stelling J. 2007 Ensemble modeling for analysis of cell signaling dynamics. Nat. Biotechnol. 25, 1001–1006. (10.1038/nbt1330) 17846631

[RSIF20090517C95] Kumar R. Clermont G. Vodovotz Y. Chow C. C. 2004 The dynamics of acute inflammation. J. Theor. Biol. 230, 145–155. (10.1016/j.jtbi.2004.04.044) 15321710

[RSIF20090517C94] Kumar A. 2006 Duration of hypotension before initiation of effective antimicrobial therapy is the critical determinant of survival in human septic shock. Crit. Care Med. 34, 1589–1596. (10.1097/01.CCM.0000217961.75225.E9) 16625125

[RSIF20090517C96] Kumareswaran K. Evans M. L. Hovorka R. 2009 Artificial pancreas: an emerging approach to treat type 1 diabetes. Expert Rev. Med. Dev. 6, 401–410. (10.1586/erd.09.23) 19572795

[RSIF20090517C97] Lee E. K. Zaider M. 2008 Operations research advances cancer therapeutics. Interfaces 38, 5–25. (10.1287/inte.1070.0327)

[RSIF20090517C98] Lee J. H. Gelormino M. S. Morari M. 1992 Model predictive control of multi-rate sampled-data systems: a state-space approach. Int. J. Control 55, 153–191. (10.1080/00207179208934231)

[RSIF20090517C99] Levy M. M. 2003 2001 sccm/esicm/accp/ats/sis international sepsis definitions conference. Crit. Care Med. 31, 1250–1256. (10.1097/01.CCM.0000050454.01978.3B) 12682500

[RSIF20090517C100] Lipiner-Friedman D. 2007 Adrenal function in sepsis: the retrospective corticus cohort study. Crit. Care Med. 35, 1012–1018. (10.1097/01.CCM.0000259465.92018.6E) 17334243

[RSIF20090517C101] Lipniacki T. Kimmel M. 2007 Deterministic and stochastic models of NF kappa B pathway. Cardiovasc. Toxicol. 7, 215–234. (10.1007/s12012-007-9003-x) 17943462

[RSIF20090517C102] Lipniacki T. Hat B. Faeder J. R. Hlavacek W. S. 2008 Stochastic effects and bistability in T cell receptor signaling. J. Theor. Biol. 254, 110–122. (10.1016/j.jtbi.2008.05.001) 18556025 PMC2577002

[RSIF20090517C103] Ljung L. 1999 System identification: theory for the user, 2nd edn Upper Saddle River, NJ: Prentice Hall PTR.

[RSIF20090517C104] Ma A. C. Kubes P. 2008 Platelets, neutrophils, and neutrophil extracellular traps (nets) in sepsis. J. Thromb. Haemost. 6, 415–420. (10.1111/j.1538-7836.2007.02865.x) 18088344

[RSIF20090517C105] Mahfouf M. Abbod M. F. Linkens D. A. 2001 A survey of fuzzy logic monitoring and control utilisation in medicine. Artif. Intell. Med. 21, 27–42. (10.1016/S0933-3657(00)00072-5) 11154872

[RSIF20090517C106] Maiti R. Agrawal N. K. 2007 Atherosclerosis in diabetes mellitus: role of inflammation. Ind. J. Med. Sci. 61, 292–306. (10.4103/0019-5359.32098) 17478962

[RSIF20090517C107] Mantzaris N. V. Daoutidis P. Srienc F. 2001 *a* Numerical solution of multi-variable cell population balance models. I. Finite difference methods Comput. Chem. Eng. 25, 1411–1440. (10.1016/S0098-1354(01)00709-8)

[RSIF20090517C108] Mantzaris N. V. Daoutidis P. Srienc F. 2001 *b* Numerical solution of multi-variable cell population balance models. II: Spectral models. Comput. Chem. Eng. 25, 1441–1462. (10.1016/S0098-1354(01)00710-4)

[RSIF20090517C109] Mantzaris N. V. Daoutidis P. Srienc F. 2001 *c* Numerical solution of multi-variable cell population balance models. III: Finite element methods. Comput. Chem. Eng. 25, 1463–1481. (10.1016/S0098-1354(01)00711-6)

[RSIF20090517C110] Marshall J. C. 2000 Clinical trials of mediator-directed therapy in sepsis: what have we learned? Intensive Care Med. 26(Suppl. 1), 75–83. (10.1007/s001340051122) 10786962

[RSIF20090517C111] Marshall J. C. 2004 Through a glass darkly: the brave new world of *in silico* modeling. Crit. Care Med. 32, 2157–2159. (10.1097/01.CCM.0000142935.34916.B5) 15483434

[RSIF20090517C112] Marshall J. C. Vincent J. L. Fink M. P. Cook D. J. Rubenfeld G. Foster D. Jr Fisher C. J. Faist E. Reinhart K. 2003 Measures, markers, and mediators: toward a staging system for clinical sepsis. A report of the Fifth Toronto Sepsis Roundtable, Toronto, Ontario, Canada, 25–26 October 2000. Crit. Care Med. 31, 1560–1567. (10.1097/01.CCM.0000065186.67848.3A) 12771633

[RSIF20090517C113] Martin G. S. Mannino D. M. Eaton S. Moss M. 2003 The epidemiology of sepsis in the United States from 1979 through 2000. N. Engl. J. Med. 348, 1546–1554. (10.1056/NEJMoa022139) 12700374

[RSIF20090517C114] Martin R. Teo K. L. 1994 Optimal control of drug administration in cancer chemotherapy. River Edge, NJ: World Scientific.

[RSIF20090517C115] Martin R. B. 1992 Optimal control drug scheduling of cancer chemotherapy. Automatica 28, 1113–1123. (10.1016/0005-1098(92)90054-J)

[RSIF20090517C116] Matzinger P. 2002 An innate sense of danger. Ann. N.Y. Acad. Sci. 961, 341–342. (10.1111/j.1749-6632.2002.tb03118.x) 12081934

[RSIF20090517C117] Meldrum K. K. Metcalfe P. Leslie J. A. Misseri R. Hile K. L. Meldrum D. R. 2006 TNF-alpha neutralization decreases nuclear factor-kappa b activation and apoptosis during renal obstruction. J. Surg. Res. 131, 182–188. (10.1016/j.jss.2005.11.581) 16412467

[RSIF20090517C118] Moore B. C. 1981 Principal component analysis in linear systems: controllability, observability, and model reduction. IEEE Trans. Aut. Control **AC-26**, 17–32.

[RSIF20090517C121] Morari M. 1994 Model predictive control: multivariable control technique of choice in the 1990s? In IFAC Symp. on Advanced Control of Chemical Processes, Kyoto, Japan, May 1994, pp. 1–16. Oxford, UK: Pergamon.

[RSIF20090517C119] Morari M. Ricker N. L. 1994 Model predictive control toolbox. Natick, MA: The MathWorks, Inc.

[RSIF20090517C120] Morari M. Zafiriou E. 1989 Robust process control. Princeton, NJ: Prentice-Hall.

[RSIF20090517C122] Muske K. R. Rawlings J. B. 1993 Model predictive control with linear models. AIChE J. 39, 262–287. (10.1002/aic.690390208)

[RSIF20090517C123] Nemzek J. A. Hugunin K. M. Opp M. R. 2008 Modeling sepsis in the laboratory: merging sound science with animal well-being. Comput. Med. 58, 120–128. PMC2703167 18524169

[RSIF20090517C124] Nucci G. Cobelli C. 2000 Models of subcutaneous insulin kinetics. A critical review. Comput. Method. Prog. Biomed. 62, 249–257. (10.1016/S0169-2607(00)00071-7) 10837910

[RSIF20090517C125] Ogunnaike B. A. 2006 Elucidating the digital control mechanism for DNA damage repair with the pp53-Mdm2 system: single cell data analysis and ensemble. J. R. Soc. Interface 3, 175–184. (10.1098/rsif.2005.0077) 16849229 PMC1618486

[RSIF20090517C126] Osuchowski M. F. Connett J. Welch K. Granger J. Remick D. G. 2009 Stratification is the key: inflammatory biomarkers accurately direct immunomodulatory therapy in experimental sepsis. Crit. Care Med. 37, 1567–1573. (10.1097/CCM.0b013e31819df06b) 19325479 PMC3670962

[RSIF20090517C127] Panacek E. A. 2004 Efficacy and safety of the monoclonal anti-tumor necrosis factor antibody f(ab’)2 fragment afelimomab in patients with severe sepsis and elevated interleukin-6 levels. Crit. Care Med. 32, 2173–2182. 15640628 10.1097/01.ccm.0000145229.59014.6c

[RSIF20090517C128] Parker R. S. Doyle F. J. III 2001 Control-relevant modeling in drug delivery. Adv. Drug Deliv. Rev. 48, 211–228. (10.1016/S0169-409X(01)00114-4) 11369083

[RSIF20090517C129] Parker R. S. Doyle F. J. III Peppas N. A. 1999 A model-based algorithm for blood glucose control in type I diabetic patients. IEEE Trans. Biomed. Eng. 46, 148–157. (10.1109/10.740877) 9932336

[RSIF20090517C130] Parker R. S. Ward J. H. Peppas N. A. Doyle F. J. III 2000 Robust H_∞_ glucose control in diabetes using a physiological model. AIChE J. 46, 2537–2549. (10.1002/aic.690461220)

[RSIF20090517C131] Pedersen M. G. Corradin A. Toffolo G. M. Cobelli C. 2008 A subcellular model of glucose-stimulated pancreatic insulin secretion. Phil. Trans. A Math. Phys. Eng. Sci. 366, 3525–3543. 10.1098/rsta.2008.0120 18653438

[RSIF20090517C132] Pfeffer K. 1993 Mice deficient for the 55 kd tumor necrosis factor receptor are resistant to endotoxic shock, yet succumb to *L. monocytogenes* infection. Cell 73, 457–467. 8387893 10.1016/0092-8674(93)90134-c

[RSIF20090517C133] Plank J. 2006 Multicentric, randomized, controlled trial to evaluate blood glucose control by the model predictive control algorithm versus routine glucose management protocols in intensive care unit patients. Diabetes Care 29, 271–276. (10.2337/diacare.29.02.06.dc05-1689) 16443872

[RSIF20090517C134] Polisetty P. K. Voit E. O. Gatzke E. P. 2006 Identification of metabolic system parameters using global optimization methods. Theor. Biol. Med. Model. 3, 4 (10.1186/1742-4682-3-4) 16441881 PMC1413512

[RSIF20090517C135] Polpitiya A. D. McDunn J. E. Burykin A. Ghosh G. K. Cobb J. P. 2009 Using systems biology to simplify complex disease: immune cartography. Crit. Care Med. 37(Suppl. 1), S16–S21. 19104218 10.1097/CCM.0b013e3181920cb0 PMC4077162

[RSIF20090517C136] Prince J. M. 2006 *In silico* and *in vivo* approach to elucidate the inflammatory complexity of cd14-deficient mice. Mol. Med. 12, 88–96. 16953560 10.2119/2006-00012.Prince PMC1578765

[RSIF20090517C137] Punzi L. Podswiadek M. Sfriso P. Oliviero F. Fiocco U. Todesco S. 2007 Pathogenetic and clinical rationale for TNF-blocking therapy in psoriatic arthritis. Autoimmune Rev. 6, 524–528. (10.1016/j.autrev.2006.12.003) 17854743

[RSIF20090517C138] Qin S. J. Badgwell T. A. 1999 An overview of nonlinear MPC applications. In Nonlinear model predictive control: assessment and future directions (eds Allgöwer F. Zheng A.). Basel, Switzerland: Birkhäuser.

[RSIF20090517C139] Quon M. J. Cochran C. Taylor S. I. Eastman R. C. 1994 Non-insulin-mediated glucose disappearance in subjects with IDDM. Discordance between experimental results and minimal model analysis. Diabetes 43, 890–896. (10.2337/diabetes.43.7.890) 8013753

[RSIF20090517C140] Ramkrishna D. 2000 Population balances: theory and applications to particulate systems in engineering. San Diego, CA: Academic Press.

[RSIF20090517C141] Rathinam M. Petzold L. R. Cao Y. Gillespie D. T. 2003 Stiffness in stochastic chemically reacting systems: the implicit tau-leaping method. J. Chem. Phys. 119, 12 784–12 794.

[RSIF20090517C142] Rawlings J. B. Meadows E. S. Muske K. R. 1994 Nonlinear model predictive control: a tutorial and survey. In IFAC Symp. on Advanced Control of Chemical Processes, Kyoto, Japan, pp. 203–214.

[RSIF20090517C143] Remick D. G. 2003 Cytokine therapeutics for the treatment of sepsis: why has nothing worked? Curr. Pharm. Des. 9, 75–82. (10.2174/1381612033392567) 12570677

[RSIF20090517C144] Resource Facility for Population Kinetics. 2008 See http://depts.washington.edu/rfpk/. (accessed 17 March 2008).

[RSIF20090517C145] Reynolds A. M. 2008 Mathematical models of acute inflammation and a full lung model of gas exchange under inflammatory stress. PhD thesis, Department of Mathematics, University of Pittsburgh, PA. See http://etd.library.pitt.edu/ETD/available/etd-06032008-223454/restricted/Reynolds_Angela_thesis_July_20.pdf.

[RSIF20090517C146] Reynolds A. M. Rubin J. Clermont G. Ermentrout B. 2006 A reduced mathematical model of the acute inflammatory response. I. Derivation of the model and analysis of anti-inflammation. J. Theor. Biol. 242, 220–236. (10.1016/j.jtbi.2006.02.016) 16584750

[RSIF20090517C148] Rivers E. Nguyen B. Havstad S. Ressler J. Muzzin A. Knoblich B. Peterson E. Tomlanovich M. 2001 Early goal-directed therapy in the treatment of severe sepsis and septic shock. N. Engl. J. Med. 345, 1368–1377. (10.1056/NEJMoa010307) 11794169

[RSIF20090517C147] Rivers E. P. Kruse J. A. Jacobsen G. Shah K. Loomba M. Otero R. Childs E. W. 2007 The influence of early hemodynamic optimization on biomarker patterns of severe sepsis and septic shock. Crit. Care Med. 35, 2016–2024. 17855815 10.1097/01.ccm.0000281637.08984.6e

[RSIF20090517C151] Roy A. 2008 Dynamic modeling of free fatty acid, glucose, and insulin during rest and exercise in insulin dependent diabetes mellitus patients. PhD thesis, Department of Chemical and Petroleum Engineering, University of Pittsburgh, PA.

[RSIF20090517C149] Roy A. Parker R. S. 2006 Dynamic modeling of free fatty acids, glucose, and insulin: An extended minimal model. Diabetes Tech. Theraput. 8, 617–626. (10.1089/dia.2006.8.617) 17109593

[RSIF20090517C150] Roy A. Parker R. S. 2007 Dynamic modeling of exercise effects on plasma glucose and insulin levels. J. Diabetes Sci. Tech. 1, 338–347. 10.1177/193229680700100305 PMC2769581 19885088

[RSIF20090517C152] Salluh J. I. Bozza P. T. 2008 Biomarkers of sepsis: lost in translation? Crit. Care Med. 36, 2192–2194. (10.1097/CCM.0b013e31817c0cd8) 18594226

[RSIF20090517C153] Sands K. E. 1997 Epidemiology of sepsis syndrome in 8 academic medical centers. Academic Medical Center Consortium Sepsis Project Working Group. JAMA 278, 234–240. (10.1001/jama.278.3.234) 9218672

[RSIF20090517C154] Sappington P. L. Yang R. Yang H. Tracey K. J. Delude R. L. Fink M. P. 2002 Hmgb1 b box increases the permeability of caco-2 enterocytic monolayers and impairs intestinal barrier function in mice. Gastroenterology 123, 790–802. (10.1053/gast.2002.35391) 12198705

[RSIF20090517C155] Sharshar T. Leclerc F. Annane D. 2006 Vasopressin in sepsis: a world of complexity. Pediatr. Crit. Care Med. 7, 281–282. (10.1097/01.PCC.0000216439.33312.F2) 16682891

[RSIF20090517C156] Sheiner L. B. Beal S. L. 1980 Evaluation of methods for estimating population pharmacokinetic parameters. I. Michaelis-Menten model: routine clinical pharmacokinetic data. J. Pharmacokinet. Biopharm. 8, 553–571. (10.1007/BF01060053) 7229908

[RSIF20090517C157] Sheiner L. B. Ludden T. M. 1992 Population pharmacokinetics/dynamics. Annu. Rev. Pharmacol. Toxicol. 32, 185–209. 1605567 10.1146/annurev.pa.32.040192.001153

[RSIF20090517C158] Shuler M. L. 1999 Single-cell models: promise and limitations. J. Biotechnol. 71, 225–228. (10.1016/S0168-1656(99)00024-3) 10483108

[RSIF20090517C159] Skogestad S. Postlethwaite I. 1996 Multivariable feedback control. New York, NY: John Wiley & Sons.

[RSIF20090517C160] Sorensen J. T. 1985 A physiologic model of glucose metabolism in man and its use to design and assess improved insulin therapies for diabetes. PhD thesis, Department of Chemical Engineering, MIT, MA.

[RSIF20090517C161] Steil G. M. Murray J. Bergman R. N. Buchanan T. A. 1994 Repeatability of insulin sensitivity and glucose effectiveness from the minimal model—implications for study design. Diabetes 43, 1365–1371. (10.2337/diabetes.43.11.1365) 7926313

[RSIF20090517C162] Sweeney D. A. Danner R. L. Eichacker P. Q. Natanson C. 2008 Once is not enough: clinical trials in sepsis. Intens. Care Med. 34, 1955–1960. (10.1007/s00134-008-1274-6) 18839140

[RSIF20090517C163] Teorell T. 1937 *a* Kinetics of distribution of substances administered to the body I. Arch. Int. Pharma. Ther. 57, 202–225.

[RSIF20090517C164] Teorell T. 1937 *b* Kinetics of distribution of substances administered to the body II. Arch. Int. Pharma. Ther. 57, 226–240.

[RSIF20090517C165] US EPA. 1984 Office of Health and Environmental Assessment by the International Life Sciences Institute. Physiological parameter values for pbpk models.

[RSIF20090517C166] Wada D. R. Ward D. S. 1994 The hybrid model: a new pharmacokinetic model for computer-controlled infusion pumps. IEEE Trans. Biomed. Eng. 41, 134–142. (10.1109/10.284924) 8026846

[RSIF20090517C167] Watson D. 2004 Cardiovascular effects of the nitric oxide synthase inhibitor ng-methyl-l-arginine hydrochloride (546c88) in patients with septic shock: results of a randomized, double-blind, placebo-controlled multicenter study (study no. 144-002). Crit. Care Med. 32, 13–20. (10.1097/01.CCM.0000104209.07273.FC) 14707555

[RSIF20090517C168] Watson R. S. Carcillo J. A. Linde-Zwirble W. T. Clermont G. Lidicker J. Angus D. C. 2003 The epidemiology of severe sepsis in children in the united states. Am. J. Resp. Crit. Care Med. 167, 695–701. (10.1164/rccm.200207-682OC) 12433670

[RSIF20090517C169] Waugh J. Perry C. M. 2005 Anakinra: a review of its use in the management of rheumatoid arthritis. BioDrugs 19, 189–202. (10.2165/00063030-200519030-00005) 15984903

[RSIF20090517C170] Weber K. M. Martin I. K. Best J. D. Alford F. P. Boston R. C. 1989 Alternative method for minimal model analysis of intravenous glucose tolerance data. Am. J. Physiol. 256, E524–535. 2650564 10.1152/ajpendo.1989.256.4.E524

[RSIF20090517C171] Weissman I. L. Shizuru J. A. 2008 The origins of the identification and isolation of hematopoietic stem cells, and their capability to induce donor-specific transplantation tolerance and treat autoimmune diseases. Blood 112, 3543–3553. (10.1182/blood-2008-08-078220) 18948588 PMC2574516

[RSIF20090517C172] Wolkenhauer O. Ullah M. Kolch W. Cho K. H. 2004 Modeling and simulation of intracellular dynamics: choosing an appropriate framework. IEEE Trans. Nanobioscience 3, 200–207. (10.1109/TNB.2004.833694) 15473072

[RSIF20090517C173] Wyss-Coray T. 2006 Inflammation in Alzheimer disease: driving force, bystander or beneficial response? Nat. Med. 12, 1005–1015. 16960575 10.1038/nm1484

[RSIF20090517C174] Yaffe M. B. Fink M. P. 2000 Cellular signaling in critical care–putting the pieces together. Crit. Care Med. 28(Suppl. 4), 1–2. (10.1097/00003246-200004001-00001) 10807311

[RSIF20090517C175] Ye X. Chu J. Zhuang Y. Zhang S. 2005 Multi-scale methodology: a key to deciphering systems biology. Front Biosci. 10, 961–965. (10.2741/1590) 15569634

[RSIF20090517C176] Yue H. Brown M. Knowles J. Wang H. Broomhead D. S. Kell D. B. 2006 Insights into the behavior of systems biology models from dynamics sensitivity and identifiability analysis: a case study of an NF-*κ* B signalling pathway. Mol. Biosyst. 2, 640–649. (10.1039/b609442b) 17216045

[RSIF20090517C177] Zafiriou E. Chiou H.-W. 1993 Output constraint softening for SISO model predictive control. In Proc. American Control Conf., San Francisco, CA, pp. 372–376. Picataway, NJ: IEEE Press.

[RSIF20090517C178] Zamamiri A. M. Zhang Y. Henson M. A. Hjortsø M. A. 2002 Dynamics analysis of an age distribution model of oscillating yeast cultures. Chem. Eng. Sci. 57, 2169–2181. (10.1016/S0009-2509(02)00109-4)

[RSIF20090517C179] Zenker S. Rubin J. Clermont G. 2007 From inverse problems in mathematical physiology to quantitative differential diagnoses. PLoS Comput. Biol. 3, e204 (10.1371/journal.pcbi.0030204). 17997590 PMC2065888

[RSIF20090517C180] Zurakowski R. Teel A. R. 2006 A model predictive control based scheduling method for HIV therapy. J. Theor. Biol. 238, 368–382. (10.1016/j.jtbi.2005.05.004) 15993900

